# ABHD17C‐Mediated S‐Depalmitoylation of BCL6B Enhances CD24 Transcription to Resist Macrophage Phagocytosis in Pancreatic Cancer

**DOI:** 10.1002/advs.75757

**Published:** 2026-05-19

**Authors:** Yalu Zhang, Di Cui, Fanzheng Meng, Xu Zhu, Liang Zhu, Liang Wu, Haifeng Hu, Jizhou Wang, Hanhui Yao, Lianxin Liu

**Affiliations:** ^1^ Department of General Surgery The First Affiliated Hospital of USTC Division of Life Science and Medicine University of Science and Technology of China Hefei Anhui China; ^2^ State Key Laboratory of Immune Response and Immunotherapy Centre For Leading Medicine and Advanced Technologies of IHM Division of Life Sciences and Medicine University of Science and Technology of China Hefei Anhui China; ^3^ Anhui Provincial Key Laboratory of Hepatopancreatobiliary Surgery Hefei Anhui China; ^4^ Anhui Provincial Clinical Research Center For Hepatobiliary Diseases Hefei Anhui China; ^5^ Fuyang Medical College Fuyang Normal University Fuyang Anhui China

**Keywords:** ABHD17C, BCL6B, CD24, macrophage, pancreatic cancer, S‐depalmitoylation

## Abstract

The phagocytic function of macrophages plays a crucial role in the innate immune response against tumors. Alpha/beta hydrolase domain‐containing protein 17C (ABHD17C) is a kind of depalmitoyltransferase and its roles in tumor immunology remain largely unclear. This study aims to investigate the role of ABHD17C in the macrophage‐mediated innate immunity in pancreatic cancer (PC). Clinically, ABHD17C is abnormally overexpressed in PC tissues and associated with poor prognosis in patients. Using orthotopic cell‐derived xenograft ‐based NSG murine models, we observed that ABHD17C‐mediated macrophage phagocytic resistance promotes tumor growth in a depalmitoylation‐dependent manner. Mechanistically, ABHD17C‐mediated depalmitoylation of B cell lymphoma 6 member B protein (BCL6B) at Cys442 impedes importin‐α/β‐mediated nuclear translocation of BCL6B and drives its ubiquitination‐dependent degradation in the cytoplasm. The deficiency of nuclear BCL6B attenuates transcriptional repression of the anti‐phagocytic signal CD24, increases its expression, enables PC cells to evade macrophage attack, and ultimately promoting PC progression. Therefore, this study elucidates the mechanism by which the depalmitoyltransferase ABHD17C protects tumor cells from macrophage phagocytosis, which highlights the therapeutic potential of targeting the ABHD17C/BCL6B/CD24 signaling axis by macrophage‐mediated innate immune pathway in PC.

## Graphical abstract

ABHD17C‐mediated depalmitoylation of BCL6B at Cys442 blocks its nuclear import and triggers ubiquitin‐dependent degradation, attenuating transcriptional repression of the anti‐phagocytic signal CD24. This mechanism enables pancreatic cancer cells to evade macrophage phagocytosis and fosters an immunosuppressive microenvironment. Targeting the ABHD17C/BCL6B/CD24 axis represents a promising innate immune checkpoint strategy for pancreatic cancer therapy.

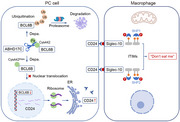

## 1 Introduction

Pancreatic cancer (PC) is one of the most aggressive and lethal malignancies, characterized by a dismal prognosis and responsible for more than 467,000 cancer‐related deaths annually worldwide [1]. Although surgical resection combined with adjuvant chemotherapy can extend long‐term survival in resectable cases, the majority of patients are diagnosed at an advanced, incurable stage, losing the opportunity for curative treatment [2]. Advanced PC is unresectable and notoriously refractory to conventional chemoradiotherapy, yielding a five‐year overall survival (OS) rate below 13% [2]. Consequently, there is an urgent unmet need to identify novel prognostic biomarkers and to develop effective therapeutic strategies for PC.

Protein function is governed by an array of post‐translational modifications —ubiquitination, methylation, phosphorylation, glycosylation and lipidation—that occur after translation in the cytoplasm [3]. Palmitoylation, a type of lipid modification, entails the covalent attachment of the 16‐carbon saturated fatty acid palmitate to specific cysteine residues via thioester (S‐palmitoylation) or, less frequently, amide (N‐palmitoylation) linkages [4]. Notably, S‐palmitoylation is dynamically reversible, and palmitoylation/depalmitoylation cycles precisely modulate protein activity, intracellular trafficking, subcellular localization, stability and downstream signaling [5, 6]. This reversible lipidation is catalyzed predominantly by the palmitoyl acyltransferases of the Asp‐His‐His‐Cys (DHHC) family, whose defining feature is a conserved DHHC catalytic domain [7]. Alpha/beta hydrolase domain‐containing protein 17 (ABHD17) family is among the few validated regulators of protein‐cysteine S‐depalmitoylating enzymes. It has been reported that the catalytic function of ABHD17 family is essential for N‐Ras depalmitoylation and re‐localization to internal cellular membranes, thereby mediating N‐Ras‐dependent cancer growth [8, 9]. Additionally, ABHD17C, a member of the ABHD17 family, inhibits ferroptosis by depalmitoylation of ALOX15B, accelerating the malignant progression of cancer [10]. However, whether depalmitoylation plays a significant regulatory role in the initiation and progression of PC remains unclear.

Tumor‐associated macrophages (TAMs) are the most abundant stromal component of the tumor microenvironment (TME) [11]. Present to widely varying degrees in solid tumors, TAMs drive tumor progression by fostering proliferation, metastasis and angiogenesis [12, 13]. They remodel the extracellular matrix through the secretion of cytokines, chemokines and growth factors [14]. Within the TME, resting macrophages (M0) can polarize into two major phenotypes: classically activated, pro‐inflammatory M1 macrophages, and alternatively activated, anti‐inflammatory M2 macrophages. Typically, macrophages are considered antitumorigenic when secreting high levels of iNOS, IFN‐γ, or TNF‐α, and pro‐tumorigenic when expressing elevated IL‐10, Arg‐1, CD163, or CD206 [15, 16]. Cancer cells can escape macrophage‐mediated clearance by overexpressing anti‐phagocytic surface molecules known as “don't eat me” signals, such as CD47 [17]. More recently, growing evidence indicates that CD24 functions as an innate immune checkpoint, acting as a novel “don't eat me” signal. Tumor‐expressed CD24 promotes immune escape by binding to the inhibitory receptor Siglec‐10 on TAMs [18]. CD24 engagement with Siglec‐10 triggers a series of inhibitory signals recruiting SHP‐1/2 phosphatases with immunoreceptor tyrosine‐based inhibition motifs (ITIMs) within the cytoplasmic domain of Siglec‐10, suppressing Toll‐like receptor‐driven inflammation and the cytoskeletal remodeling necessary for macrophage phagocytosis [18, 19]. Therefore, elucidating the precise molecular mechanisms of CD24 in PC may contribute to the development of novel therapeutic strategies.

In the present study, we show that ABHD17C is aberrantly overexpressed in PC and serves as a predictor of poor prognosis. Mechanistically, ABHD17C catalyzes the depalmitoylation of BCL6B at Cys442, which inhibits its importin‐α/β‐mediated nuclear translocation and facilitates its ubiquitin‐proteasome‐mediated degradation. This process attenuates BCL6B‐mediated transcriptional repression of CD24, thereby conferring resistance to TAM phagocytosis in vivo. Together, our findings delineate an ABHD17C/BCL6B/CD24 axis that drives PC progression in a depalmitoylation‐dependent manner.

## 2 Results

### 2.1 Aberrant ABHD17C Upregulation Predicts Unfavorable Prognosis in PC

**FIGURE 1:**
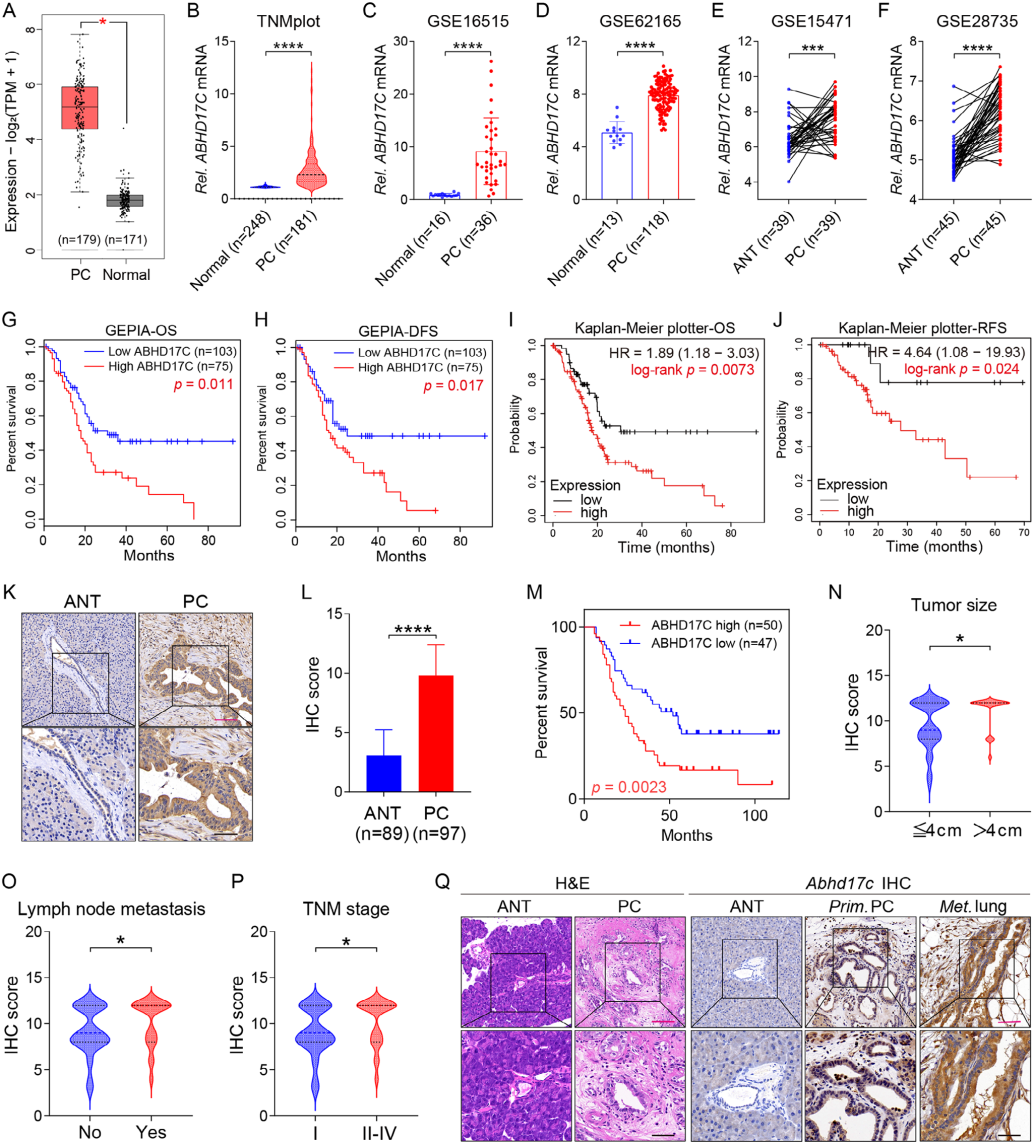
Aberrant ABHD17C upregulation predicts unfavorable prognosis in PC. (A) The expression of ABHD17C between pancreatic cancer (PC) (*n* = 179) and normal pancreatic tissues (*n* = 171) was analyzed by the GEPIA web tool. (B) The transcriptional level of ABHD17C between PC (*n* = 181) and normal pancreatic tissues (*n* = 248) was explored using the TNMplot database. (C, D) Unpaired datasets GSE16515 and GSE62165 from the GEO database were used to compare the mRNA level of ABHD17C between PC and normal pancreatic tissues. GSE16515: Normal (*n* = 16); PC (*n* = 36). GSE62165: Normal (*n* = 13); PC (*n* = 118). (E, F) Paired datasets GSE15471 (*n* = 39) and GSE28735 (*n* = 45) were used to further verify the mRNA level of ABHD17C between PC and adjacent normal tissue (ANT). (G, H) ABHD17C‐related overall survival (OS) and disease‐free survival (DFS) in patients with PC were analyzed by the GEPIA, with the conditions set as “Cutoff‐High vs. Cutoff‐Low: 42 vs. 42”. Grouping: low ABHD17C (*n* = 103); high ABHD17C (*n* = 75). (I, J) The Kaplan–Meier plotter based on publicly available data was employed to evaluate the prognostic value of ABHD17C expression for OS and relapse‐free survival (RFS) in a cohort of patients with PC. OS grouping: low ABHD17C (*n* = 62); high ABHD17C (*n* = 115). RFS grouping: low ABHD17C (*n* = 16); high ABHD17C (*n* = 53). (K) Representative images of immunohistochemistry (IHC) analysis for ABHD17C on tissue microarray (TMA). Red bar: 100 µm; black bar: 50 µm. (L) IHC scores of ABHD17C were assessed in TMA containing 89 samples of ANT and 97 samples of PC tissue. (M) ABHD17C‐related OS in a cohort of patients with PC from the independent samples of TMA. Grouping: ABHD17C low (*n* = 50); ABHD17C high (*n* = 47). (N–P) Compare the differences in IHC scores across various clinical parameters derived from TMA, including tumor size, lymph node metastasis, and TNM staging. Tumor size: ≦ 4 cm (*n* = 79); > 4 cm (*n* = 18). lymph node metastasis: No (*n* = 37); Yes (*n* = 60). TNM staging: I (*n* = 33); II‐IV (*n* = 64). (Q) Representative hematoxylin and eosin (HE) staining of PC and ANT, and IHC analysis of Abhd17c expression in ANT, orthotopic PC, and lung metastases in a DMBA‐induced spontaneous mouse model of PC. Red bar: 100 µm; black bar: 50 µm. Abbreviation: TPM, transcripts per million; *Rel*., relative; PC, pancreatic cancer; ANT, adjacent normal tissue; GEPIA, Gene Expression Profiling Interactive Analysis; OS, overall survival; DFS, disease‐free survival; RFS, relapse‐free survival; IHC, immunohistochemistry; TNM, tumor, node, and metastasis; H&E, hematoxylin and eosin; *Prim*., primary; *Met*., metastatic. Each error bar in A‐F, L, N‐P represents the mean ± SD; ^*^ *p* < 0.05, ^***^ *p* < 0.001, ^****^ *p* < 0.0001. Statistical analysis was performed using a one‐tailed Mann‐Whitney *U* test (A–D, L, N–P), a paired two‐tailed Student's *t* test (E, F), or the log‐rank test (G–J, M).

To investigate the expression level of ABHD17C in PC, we first investigated public databases. Gene Expression Profiling Interactive Analysis (GEPIA) database showed that the transcriptional level of ABHD17C was higher in PC compared to normal pancreas (Figure 1A). Unpaired data from the TNMplot, GSE16515, and GSE62165 datasets (Figure 1B–D), along with paired datasets from GSE15471 and GSE28735 (Figure 1E,F), further confirmed that ABHD17C mRNA levels were significantly elevated in PC tissues relative to normal pancreatic tissues from healthy controls or adjacent normal tissues (ANTs) from PC patients. The Human Protein Atlas database displayed that the protein level of ABHD17C was upregulated in PC tissues compared with normal pancreatic tissues (Figure S1A). Subsequently, analysis of single‐cell RNA sequencing data from PC tissues revealed that ABHD17C was specifically highly expressed in malignant PC cells among the heterogeneous cellular components, including acinar, endocrine, stellate, immune cells, and fibroblasts (Figure S1B). In contrast, its expression was nearly absent in normal pancreatic ductal cells (Figure S1B). GEPIA and Kaplan–Meier plotter databases indicated that patients with high ABHD17C expression had shorter OS and disease/relapse‐free survival (DFS/RFS) (Figure 1G–J). Tumor Immune Estimation Resource (TIMER), LinkedOmics, and Tumor Immune System Interaction Database (TISIDB) also implicated that high ABHD17C expression was associated with a poorer prognosis and shorter OS in patients with PC (Figure S1C–E). Besides, GEPIA database showed that the expression level of ABHD17C varied significantly across different pathological stages of PC (Figure S1F). According to LinkedOmics, elevated ABHD17C expression was associated with larger tumor size and more advanced disease stage in PC (Figure S1G,H). More importantly, the GSE71729 dataset indicated that ABHD17C expression was higher in liver and lung metastases of PC than in the primary tumors (Figure S1I).

To validate these findings, we collected 89 samples of ANT and 97 samples of PC tissue, and constructed a PC tissue microarray (TMA) for immunohistochemical (IHC) staining (Figure 1K). Double‐blinded IHC scoring by senior pathologists confirmed that ABHD17C expression was higher in PC tissues than in ANTs (Figure 1L). Furthermore, high ABHD17C expression was identified as a negative prognostic factor in PC patients (Figure 1M), with median OS times of 24 and 52 months in the high and low expression groups, respectively. Additionally, higher ABHD17C expression was observed in PC tumors with larger size (≥ 4 cm), lymph node metastasis, and advanced TNM stages (Figure 1N–P; Table S1). However, no significant associations were found between ABHD17C level and other clinical parameters in PC patients, including age, gender, histological grade, CA199 level, perineural invasion, and macrovascular invasion (Table S1). To further investigate the expression level of ABHD17C, we successfully established a spontaneous mouse PC model by orthotopically embedding the potent carcinogen dimethylbenzanthracene (DMBA) into the pancreas of C57BL/6 mice, with model validation confirmed by hematoxylin and eosin (HE) staining (Figure 1Q). The results verified that ABHD17C expression is upregulated in pancreatic tissues upon malignant transformation, and its expression was further elevated in lung metastases compared to primary tumors (Figure 1Q).

The univariate analysis demonstrated that ABHD17C expression, histological grade, tumor size, lymph node metastasis, TNM stage, and CA199 were associated with the prognosis of patients with PC (Table S2). These variables were subsequently included in a Cox proportional hazards regression model. Multivariate analysis revealed that ABHD17C expression, histological grade, tumor size, and CA199 were the independent adverse prognostic factor (Table S2). Furthermore, subgroup analysis of patients with PC indicated that high ABHD17C expression was associated with a worse prognosis in subgroups of patients with the following characteristics: Age ≥ 60 years, male, histological G2‐3 grade, tumor size < 4 cm, perineural invasion‐negative, macrovascular invasion‐negative, and CA199‐normal (Figure S2 and Table S3).

### 2.2 ABHD17C Confers Macrophage Phagocytic Resistance via Potentiation of CD24/Siglec‐10 Signaling

In the DMBA‐induced immunocompetent spontaneous PC mouse model, substantial macrophage infiltration in both primary and lung metastatic lesions (Figure S3A, left panel). Similarly, significant TAM infiltration was observed in human PC tissues (Figure S3A, right panel). Analysis using the immune infiltration module of the TIMER database indicated an inversely correlation between ABHD17C expression and macrophage infiltration in PC tissues (Figure S3B). These findings hinted that ABHD17C might be involved in regulating the TME of PC, particularly in modulating macrophages. By querying different databases (LinkedOmics, GEPIA, and TIMER), we unexpectedly found that ABHD17C expression was significantly positively correlated with the “don't eat me” signal CD24 (Figure S3C–E). Based on these results, we hypothesized that ABHD17C may enhance the anti‐phagocytic signaling of CD24, thereby enabling evasion of macrophage phagocytosis in TME of PC.

**FIGURE 2:**
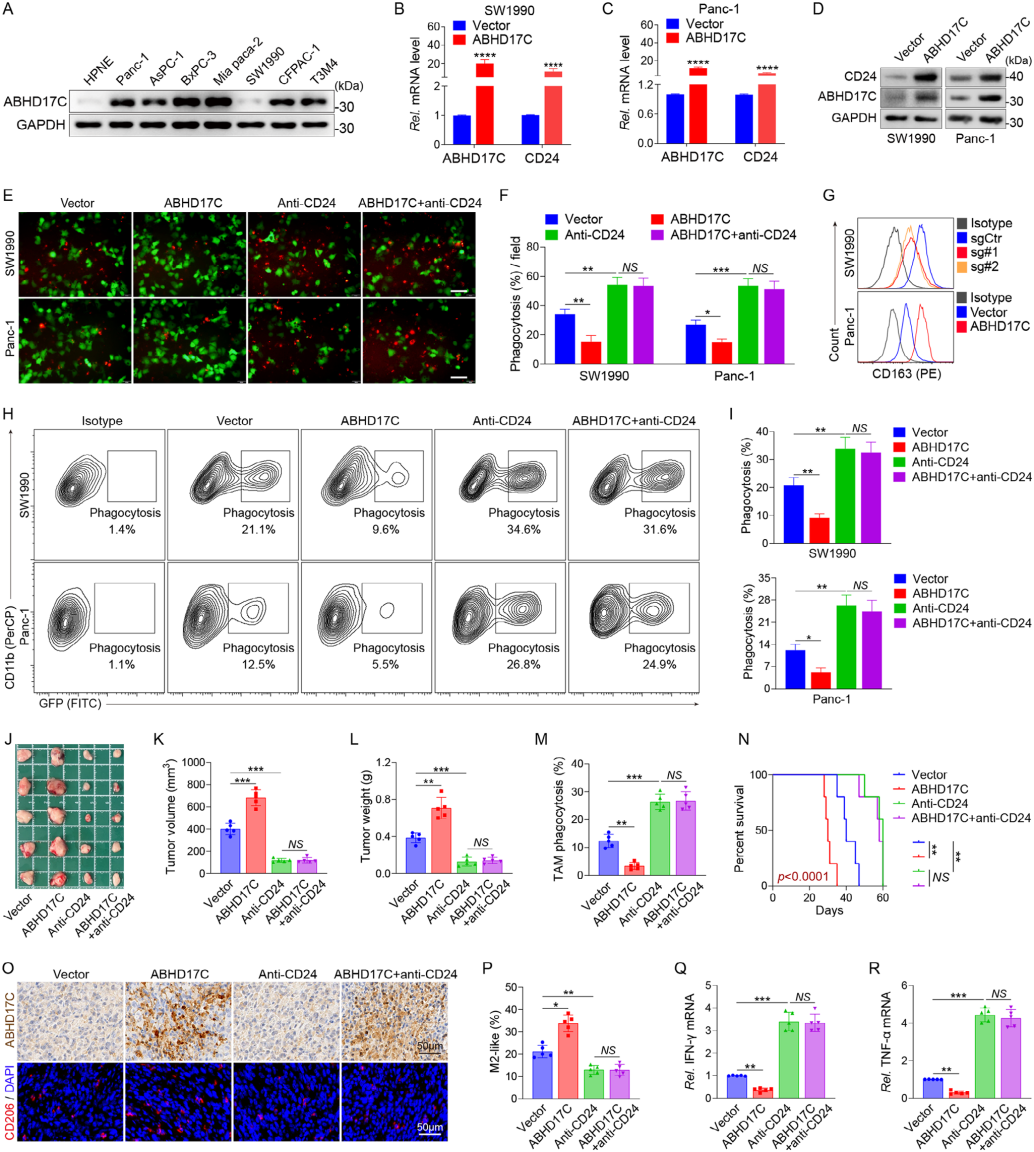
ABHD17C confers macrophage phagocytic resistance via potentiation of CD24‐Siglec‐10 signaling. (A) Western blot analysis of ABHD17C protein levels in the immortalized pancreatic ductal epithelial cell line HPNE and seven PC malignant cell lines. (B–D) The mRNA and protein levels of ABHD17C and CD24 were measured in SW1990 and Panc‐1 cells following transfection with ABHD17C‐overexpressing plasmids. (E, F) Representative images of phagocytosis assays. Peripheral blood mononuclear cell (PBMC)‐derived macrophages were co‐cultured with pHrodo‐red^+^ GFP^+^ SW1990 or Panc‐1 cells. Tumor cells had been transfected with ABHD17C‐plasmid or empty vector and pre‐incubated with IgG control or anti‐CD24 mAb. Red puncta inside macrophages indicate engulfed tumor cells. Scale bar, 100 µm. (G) An indirect co‐culture system of macrophages with SW1990 cells (ABHD17C‐KO) or Panc‐1 cells (ABHD17C‐OE) was employed to evaluate the role of ABHD17C in macrophage polarization, which was assessed via CD163 expression by flow cytometry. (H, I) Flow cytometry (FCM)‐based measurement showing phagocytosis of SW1990 or Panc‐1 cells, transfected with ABHD17C‐plasmid or empty vector, and pre‐treated with IgG control or anti‐CD24 mAb. Cells double‐positive for CD11b and GFP were defined as engulfed cells. (J) Representative images of orthotopic pancreatic cell‐derived xenograft (CDX) model. SW1990 cells infected with the indicated lentivirus were injected into the pancreatic tail of NSG mice, and treated with IgG control or anti‐CD24 mAb by intraperitoneal injection. *n* = 5 mice per group. Tumors were excised, photographed, and weighed at day 28. (K, L) Excised tumor volume (mm^3^) and mass (g) were measured. (M) Tumor masses were digested to single‐cell suspensions and analyzed by flow cytometry. The frequency of phagocytosis events within total tumor‐associated macrophages (TAMs) was compared across groups. (N) An independent cohort of NSG mice was subjected to survival analysis following orthotopic inoculation with the indicated tumors and treatments. *n* = 5 mice per group. (O) Representative images of immunohistochemistry (IHC) staining for ABHD17C and immunofluorescence (IF) staining for CD206 in CDX tumors from NSG mice with indicated treatments. Scale bar, 50 µm. (P) The frequency of CD206‐positive (M2‐like) TAMs was determined among total TAMs. (Q, R) The mRNA level of TNF‐α and IFN‐γ was detected in tumor masses across different groups. Abbreviation: *Rel*., relative; TAM, tumor‐associated macrophage; DAPI, 4’,6‐diamidino‐2‐phenylindole. Data are the mean ± SD of *n* = 3 (B, C, F, I) and *n* = 5 (K–N, P–R) independent biological replicates; ^*^ *p* < 0.05, ^**^ *p* < 0.01, ^***^ *p* < 0.001, ^****^ *p* < 0.0001; *NS*, not significant. Statistical analysis was performed using an unpaired two‐tailed Student's *t* test (B, C), or a one‐way ANOVA with Tukey's post‐hoc test (F, I, K–M, P–R), and survival curves were compared using the Log‐rank (Mantel‐Cox) test (N).

To investigate the biological roles of ABHD17C in PC cells, we first analyzed the protein expression profile of ABHD17C in the normal pancreatic ductal epithelial cell HPNE and seven PC malignant cell lines. The results showed that ABHD17C expression was higher in all PC cell lines compared to HPNE, except for SW1990 (Figure 2A; Figure S3F). For gain‐ and loss‐of‐function studies, stable ABHD17C overexpression (OE) was established in SW1990 and Panc‐1 cells using lentiviral transduction; concurrently, ABHD17C knockout (KO) clones (sg#1 and sg#2) were constructed in Mia Paca‐2 cells using CRISPR‐Cas9 technology. ABHD17C‐OE elevated CD24 mRNA abundance (Figure 2B,C), whereas ABHD17C‐KO markedly reduced CD24 transcript levels (Figure S3G). These transcriptional changes were consistently reflected at the protein level (Figure 2D; Figure S3H,I). To quantify phagocytic clearance by fluorescence microscopy, GFP^+^ PC cells were pre‐labeled with pHrodo Red, a dye that fluoresces upon acidification in phagolysosomes [20], and directly co‐cultured with macrophages derived from peripheral blood mononuclear cells (PBMCs). Over 24 h, we observed that ABHD17C‐KO cells were more readily engulfed and degraded in the low‐pH phagolysosome as compared to control cells (Figure S3J). In contrast, ABHD17C‐OE cells exhibited a robust resistance to phagocytosis by macrophages (Figure 2E,F), and blocking with anti‐CD24 monoclonal antibody (mAb) effectively promoted macrophage phagocytosis and eliminated the anti‐phagocytic effect induced by ABHD17C (Figure 2E,F). Flow cytometry (FCM)‐based analysis further confirmed these findings (Figure S3K; Figure 2H,I). Siglec‐10 on the surface of macrophages serves as a key ligand for CD24, and the CD24/Siglec‐10 signaling plays a critical anti‐phagocytic role in tumor immunity [18]. Similarly, blocking anti‐Siglec‐10 also facilitated macrophage phagocytosis and abolished the ABHD17C‐mediated anti‐phagocytic effect (Figure S3L,M). The gating scheme for in vitro phagocytosis is shown in Figure S4A. Additionally, given the important regulatory role of M1/M2 polarization in the TME of PC [21], PBMCs were induced to differentiate into M0 macrophages. Using an indirect co‐culture system of PC cells and macrophages, we found that PC cells induced the polarization of M0 macrophages toward the M2‐type (Figure 2G; Figure S4B). This polarization process was inhibited by ABHD17C KO but promoted by ABHD17C overexpression (Figure 2G; Figure S4B). These data indicated that ABHD17C protects PC cells from macrophage attack by the “don't eat me” signal CD24, and drives macrophage polarization toward the immunosuppressive M2‐type.

To investigate whether the protection against phagocytosis conferred by ABHD17C could be recapitulated in vivo, orthotopic cell‐derived xenograft (CDX) model of the pancreas was constructed using NOD.Cg‐*Prkd*c^scid^ *Il2rg* ^em1Smoc^ (NSG) mice [18]. At 28 days post‐engraftment, we observed that the growth of ABHD17C‐OE tumors was increased compared to the control group, and this pro‐tumor effect could be blocked by the anti‐CD24 mAb (Figure 2J–L). Importantly, altered expression of ABHD17C did not affect cell‐autonomous proliferation of PC cells in vitro (Figure S4C). FCM analysis demonstrated that ABHD17C‐OE tumors exhibited reduced levels of in vivo phagocytosis by infiltrating TAMs as compared to the control tumors, whereas CD24 blockade abrogated the anti‐phagocytic role mediated by ABHD17C (Figure 2M). The gating scheme for in vivo phagocytosis was shown in Figure S4D. Driven by attenuated phagocytic clearance, the accelerated tumor growth imposed a significant survival disadvantage on mice bearing ABHD17C‐OE tumors, while CD24 blockade effectively reversed the ABHD17C‐mediated survival disadvantage (Figure 2N). IHC staining confirmed the protein expression level of ABHD17C within tumor tissues across different groups (Figure 2O). Moreover, TAMs infiltrating the ABHD17C‐OE tumors possessed a more immunosuppressive phenotype, while CD24 blockade inhibited the ABHD17C‐induced M2‐like polarization of TAMs (Figure 2O,P; Figure S4D). ABHD17C‐OE tumors exhibited decreased levels of the inflammatory cytokines IFN‐γ and TNF‐α compared to the control group (Figure 2Q,R). In contrast, blockade of CD24 signaling effectively reversed this suppression of pro‐inflammatory factors induced by ABHD17C within the tumor microenvironment (Figure 2Q,R). Altogether, these results suggested that ABHD17C depends on CD24/Siglec‐10 signaling to evade TAM phagocytosis and promote immunosuppression in the tumor microenvironment.

### 2.3 ABHD17C Inhibits Macrophage Phagocytosis in a Depalmitoylation‐Dependent Manner

**FIGURE 3:**
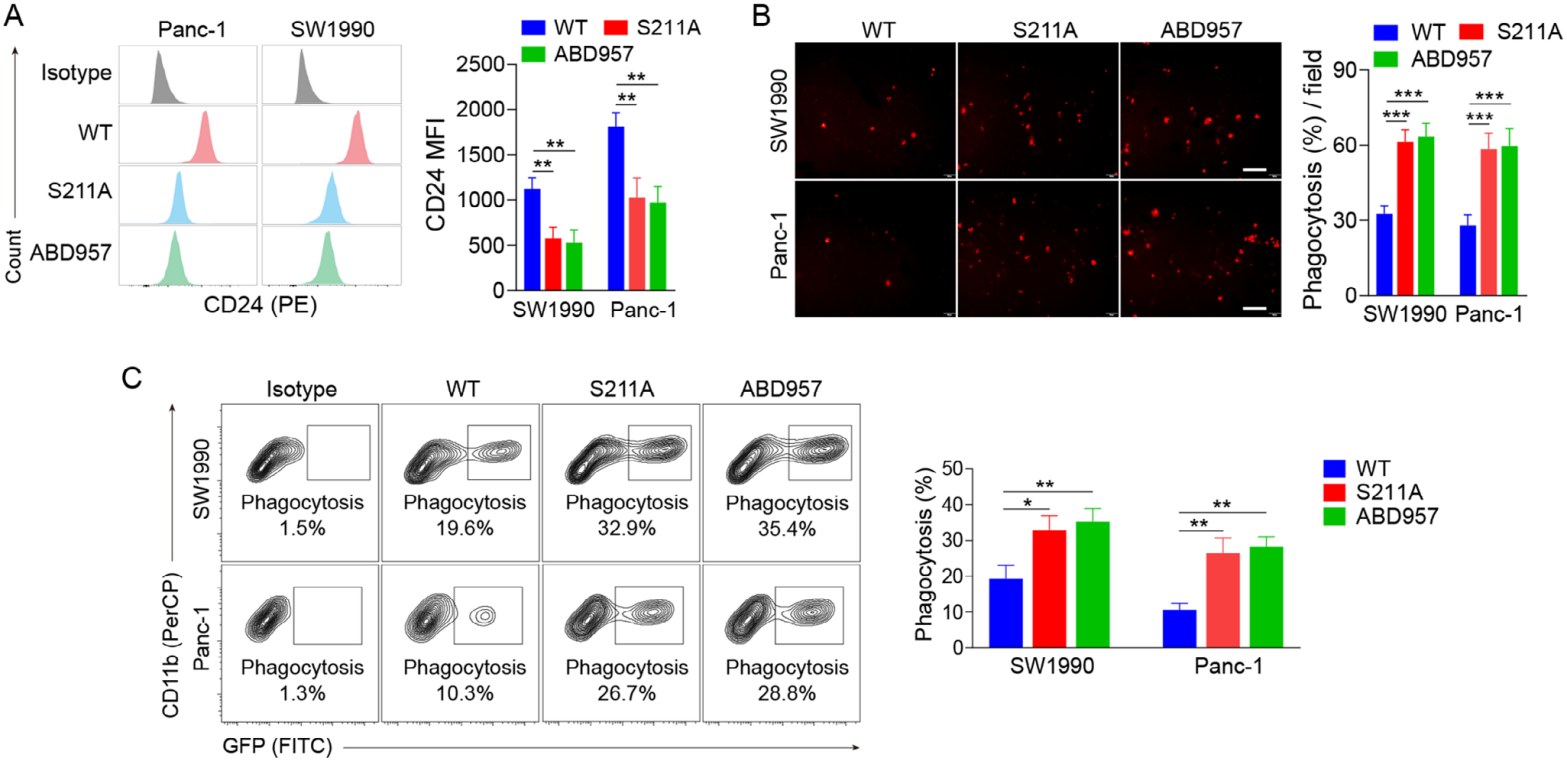
ABHD17C inhibits macrophage phagocytosis in a depalmitoylation‐dependent manner. (A) Flow cytometric analysis of CD24 expression in SW1990 and Panc‐1 cells transfected with ABHD17C WT or S211A mutation, or with ABHD17C WT combined with ABD957 treatment. (B) Representative phagocytosis images of macrophages co‐cultured with pHrodo Red‐labeled SW1990 or Panc‐1 cells, which were transfected with ABHD17C WT or S211A alone, or with ABHD17C WT combined with ABD957 treatment. Red puncta inside macrophages indicate engulfed tumor cells. Scale bar, 100 µm. (C) The frequency of phagocytosis events out of all macrophages among different groups. CD11b^+^ GFP^+^ cells were defined as engulfed cells. Abbreviation: WT, wild‐type; MFI, mean fluorescence intensity. Data are the mean ± SD of *n* = 3 (A–C) independent biological replicates; ^*^ *p* < 0.05, ^**^ *p* < 0.01, ^***^ *p* < 0.001. Statistical analysis was performed using a one‐way ANOVA with Dunnett's post‐hoc test (A–C).

Considering the function of ABHD17C as a depalmitoyl acyltransferase [8, 22], we wondered whether the phagocytosis‐inhibiting capacity of ABHD17C is dependent on its depalmitoylation activity. A previous study reported that a residue, Ser211, in the acyl binding cavity of ABHD17 family are essential for its catalytic activity [8]. Therefore, we constructed a ABHD17C mutant (S211A) without significant catalytic activity and then transfected SW1990 and Panc‐1 cells with plasmids expressing wild‐type (WT) ABHD17C and the mutant. Loss of ABHD17C depalmitoylation activity due to the S211A mutation decreased CD24 expression and promoted phagocytosis of macrophages on PC cells (Figure 3A–C). Furthermore, ABD957 is a potent, selective, and covalent inhibitor of the ABHD17 family of depalmitoylases [9]. Similarly, ABD957 treatment of PC cells with ABHD17C‐ WT reduced the level of the anti‐phagocytic signal CD24 and enhanced macrophage phagocytosis (Figure 3A–C). Collectively, these findings suggested that ABHD17C suppresses macrophage phagocytosis in a depalmitoylation‐dependent manner.

### 2.4 BCL6B is a Functional Downstream Substrate of ABHD17C in Regulating Phagocytosis

**FIGURE 4:**
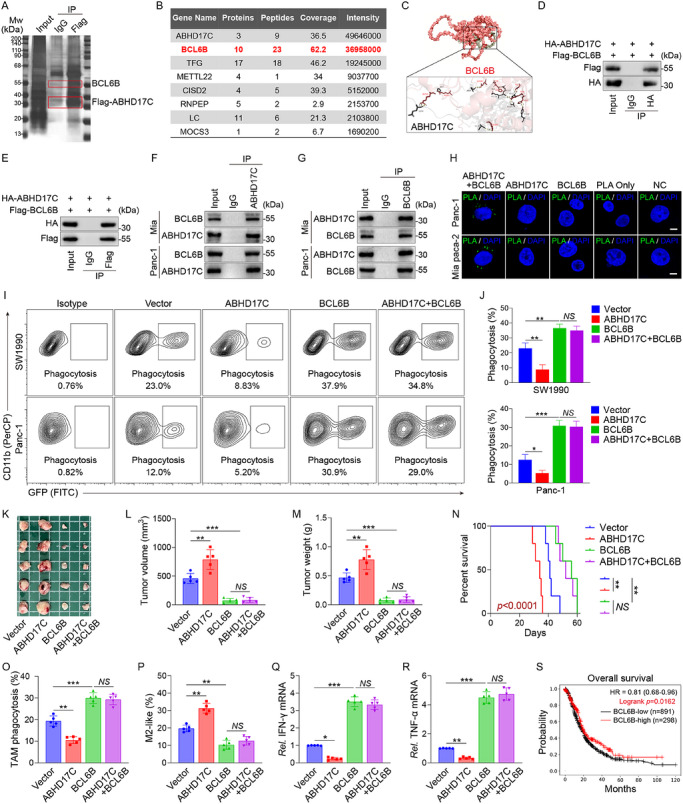
BCL6B is a functional downstream substrate of ABHD17C in regulating phagocytosis. (A) Silver staining of Flag‐ABHD17C‐immunoprecipitation following gel electrophoresis. The red frame indicated the BCL6B and Flag‐ABHD17C predicted based on molecular weight. (B) Mass‐spectrometric analysis of Flag‐ABHD17C immunoprecipitates from Panc‐1 cells. (C) Molecular docking analysis of the binding interface between ABHD17C (gray) and BCL6B (red). The detailed view of the binding sites and critical residues in the interaction is shown. (D, E) Western blot analysis of ectopically expressed HA‐ABHD17C and Flag‐BCL6B following reciprocal immunoprecipitation with anti‐HA and anti‐Flag antibodies in 293T cells. (F, G) Western blot analysis of endogenous ABHD17C and BCL6B proteins following reciprocal immunoprecipitation with anti‐ABHD17C and anti‐BCL6B antibodies in Mia paca‐2 and Panc‐1 cells. (H) The proximity ligation assay (PLA) was performed with the indicated antibodies and a negative control (NC) to verify the interaction between ABHD17C and BCL6B in Mia PaCa‐2 and Panc‐1 cells. Scale bar, 5 µm. (I, J) Flow cytometric analysis of phagocytosis in SW1990 and Panc‐1 cells upon ABHD17C and/or BCL6B transfection. CD11b and GFP double‐positive cells were classified as engulfed. (K) Representative images of orthotopic pancreatic cell‐derived xenografts (CDXs) in NSG mice engrafted with Panc‐1 cells transfected with ABHD17C or BCL6B, or co‐transfected with both. *n* = 5 mice per group. Tumors were excised, photographed, and weighed 28 days post‐injection. (L, M) Excised tumor volume (mm^3^) and mass (g) were determined. (N) Kaplan–Meier survival analysis of an independent NSG mouse cohort following orthotopic inoculation with the indicated tumor cells. *n* = 5 mice per group. (O) The frequency of phagocytosis events out of all tumor‐associated macrophages (TAMs) among different groups after engraftment. (P) The proportion of CD206^+^ (M2‐like) TAMs within the total TAM population was quantified. (Q, R) The mRNA level of TNF‐α and IFN‐γ was measured in mouse tumor tissues across different groups. (S) The online public database Kaplan–Meier plotter was employed to explore the impact of BCL6B expression on overall survival in patients with PC. Grouping: BCL6B‐low (*n* = 891), BCL6B‐high (*n* = 298). Abbreviation: Mw, molecular weight; IP, immunoprecipitation; PLA, proximity ligation assay; NC, negative control; DAPI, 4’,6‐diamidino‐2‐phenylindole; TAM, tumor‐associated macrophage; *Rel*., relative. Data are the mean ± SD of *n* = 3 (J) and *n* = 5 (L–R) independent biological replicates; ^*^ *p* < 0.05, ^**^ *p* < 0.01, ^***^ *p* < 0.001; *NS*, not significant. Statistical analysis was performed using a one‐way ANOVA with Tukey's post‐hoc test (J, L, M, O–R), and survival curves were compared using the Log‐rank (Mantel‐Cox) test (N).

Sliver staining for gel electrophoresis of Flag‐ ABHD17C‐immunoprecipitation in Panc‐1 cells was performed to display potential interacting proteins based on molecular weight (Figure 4A). To elucidate the mechanisms of ABHD17C in anti‐phagocytosis, we employed immunoprecipitation coupled with tandem mass spectrometry (IP‐MS) to identify its candidate binding partners. The IP‐MS assay revealed that there are potential interactions between ABHD17C and BCL6B (Figure 4B). The protein‐protein interaction between ABHD17C and BCL6B was predicted and visualized by molecular docking based on their structural models (Figure 4C). Co‐immunoprecipitation (Co‐IP) assays in 293T cells co‐transfected with HA‐ABHD17C and Flag‐BCL6B warranted the interaction of exogenously expressed ABHD17C with BCL6B (Figure 4D,E). Then, the interaction of ABHD17C with BCL6B in Mia paca‐2 and Panc‐1 cells will also be verified (Figure 4F,G). The proximity ligation assay (PLA) further confirmed the transient interaction of endogenous ABHD17C with BCL6B in the cytoplasm of Mia paca‐2 and Panc‐1 cells (Figure 4H). Given that ABHD17C acts as a depalmitoyl acyltransferase, we speculated that BCL6B might be an important substrate for ABHD17C. As expected, ABHD17C‐KO significantly elevated the expression of BCL6B, while ABHD17C‐OE strongly reduced the protein level of BCL6B, indicating BCL6B is a critical substrate of ABHD17C (Figure S4E). Next, we further explored whether ABHD17C inhibits macrophage phagocytosis via BCL6B. Fluorescence microscopy showed that pHRodo‐Red^+^ PC cells transfected with BCL6B plasmids were more readily engulfed into the low pH phagolysosome of macrophages, and BCL6B‐OE effectively reversed ABHD17C‐mediated phagocytic resistance of macrophages (Figure S4F). Analogously, FCM‐based measurements of phagocytosis exhibited a robust increase in the phagocytic uptake of BCL6B‐overexpressing cells as compared to vector control, and BCL6B‐OE completely abolished the anti‐phagocytic effect exerted by ABHD17C (Figure 4I,J; Figure S4A).

To further investigate whether ABHD17C promotes PC progression via BCL6B in vivo, a panel of in situ pancreatic CDX models was established in NSG mice using Panc‐1 cells transfected with ABHD17C and/or BCL6B plasmids (Figure 4K). Overexpression of BCL6B resulted in significant reduction of tumor volume and weight as compared to vector control, while BCL6B‐OE abolished ABHD17C‐mediated tumor growth and progression (Figure 4L,M). The heightened intra‐tumoral phagocytic clearance conferred a significant survival benefit for mice engrafted with BCL6B‐overexpressing tumors, and BCL6B‐OE fully rescued the adverse effects of ABHD17C on survival (Figure 4N,O; Figure S4D). Furthermore, we found a reduced polarization of M2‐type TAMs within BCL6B‐OE tumor microenvironment as compared to the vector control, and BCL6B‐OE reversed the ABHD17C‐driven immunosuppressive phenotype in TAMs (Figure 4P; Figure S4D). BCL6B‐OE tumors displayed sharply elevated levels of the anti‐tumoral cytokines IFN‐γ and TNF‐α relative to vector controls, and overexpression of BCL6B could abrogate ABHD17C‐induced suppression of IFN‐γ and TNF‐α (Figure 4Q,R). Survival analysis from Kaplan–Meier plotter showed that PC patients with high BCL6B expression had better OS than those with low BCL6B levels (Figure 4S). Taken together, these results suggested that BCL6B functions as the key downstream substrate of ABHD17C to mediate its macrophage polarization and phagocytosis resistance.

### 2.5 ABHD17C Regulates the Depalmitoylated Process of BCL6B at Cysteine 442

**FIGURE 5:**
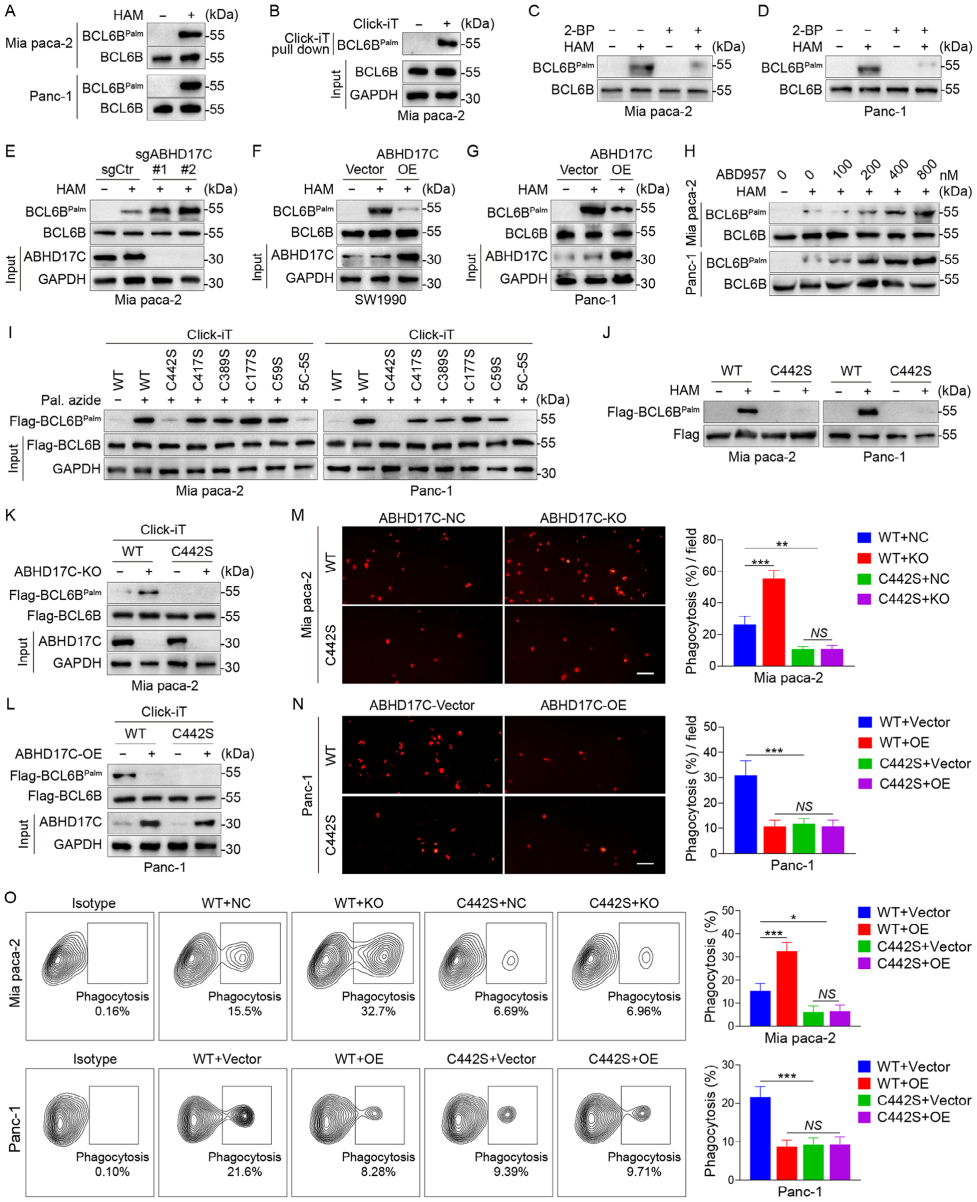
ABHD17C regulates the depalmitoylated process of BCL6B at cysteine 442. (A) BCL6B palmitoylation was detected in Mia paca‐2 and Panc‐1 cells using streptavidin‐HRP/anti‐BCL6B after immunoprecipitation with anti‐BCL6B and ABE assay. (B) Mia PaCa‐2 cells, treated with or without ALK‐C16, were harvested and subjected to a Click‐iT reaction followed by streptavidin pulldown. (C, D) BCL6B palmitoylation was assessed in Mia paca‐2 and Panc‐1 cells with or without 2‐BP treatment using streptavidin‐HRP/anti‐BCL6B after immunoprecipitation with anti‐BCL6B and ABE assay. (E) BCL6B palmitoylation was detected in Mia paca‐2 cells with or without ABHD17C‐KO using streptavidin‐HRP/anti‐BCL6B after immunoprecipitation with anti‐BCL6B and acyl‐biotin exchange (ABE) assay. (F, G) BCL6B palmitoylation was evaluated in SW1990 and Panc‐1 cells with or without ABHD17C overexpression using the ABE assay. (H) BCL6B palmitoylation was detected in Mia paca‐2 and Panc‐1 cells with the indicated concentration gradient of ABD957 using the ABE assay. (I) Click‐iT assays showing BCL6B palmitoylation status in Mia paca‐2 or Panc‐1 cells transfected with Flag‐BCL6B or its multiple mutants as indicated. Substitution of cysteine with serine at five candidate sites, or mutation of all five cysteines to serines (5C‐5S), in BCL6B. (J) Flag‐BCL6B palmitoylation was detected in Mia paca‐2 or Panc‐1 cells infected with Flag‐BCL6B WT/C442S plasmids using streptavidin HRP/anti‐ BCL6B after immunoprecipitation with anti‐Flag and ABE assay. (K, L) Flag‐tagged BCL6B WT/C442S were transfected in ABHD17C‐KO Mia paca‐2 cells or in ABHD17C‐OE Panc‐1 cells. Western blot analysis assessed the levels of Flag‐ BCL6B and its palmitoylation via the Click‐iT assay across the indicated groups. (M, N) Phagocytosis assays were performed by co‐culturing peripheral blood mononuclear cell (PBMC)‐derived macrophages with pHrodo‐red^+^ ABHD17C‐KO Mia paca‐2 or ABHD17C‐OE Panc‐1 cells infected with Flag‐BCL6B WT/C442S. Red puncta inside macrophages indicate engulfed Mia paca‐2 or Panc‐1 cells. Scale bar, 100 µm. (O) FCM assays were performed by co‐culturing macrophages with ABHD17C‐KO Mia paca‐2 or ABHD17C‐OE Panc‐1 cells infected with Flag‐BCL6B WT/C442S. CD11b^+^ GFP^+^ cells were defined as engulfed cells. Abbreviation: HAM, hydroxylamine‐assisted method; Ctr, control; Palm, palmitoylation; Pal.‐Azide, Azide‐labeled palmitic acid; 2‐BP, 2‐bromopalmitate; OE, overexpression; KO, knockout. Data are the mean ± SD of *n* = 3 (M–O) independent biological replicates; ^*^ *p* < 0.05, ^**^ *p* < 0.01, ^***^ *p* < 0.001; *NS*, not significant. Statistical analysis was performed using a one‐way ANOVA with Tukey's post‐hoc test (M–O).

As an a depalmitoyl acyltransferase [8, 22], ABHD17C most likely depalmitoylates its downstream substrate BCL6B. To determine whether BCL6B is palmitoylated, we detected palmitoylation level of endogenous BCL6B in PC cells by acyl‐biotin exchange (ABE) and click chemistry (Figure 5A,B). Besides, the omission of hydroxylamine (HAM) treatment abolished the palmitoylation of BCL6B, and BCL6B palmitoylation levels were significantly reduced after treatment with the palmitoylation inhibitor 2‐bromopalmitate (2‐BP), confirming that BCL6B is S‐palmitoylated through thioester bonds (Figure 5C,D). As expected, knockout of ABHD17C increased the level of palmitoylated BCL6B (Figure 5E). In contrast, ABHD17C overexpression decreased the level of palmitoylated BCL6B (Figure 5F,G). ABD957 could enhance the palmitoylation level of BCL6B in a dose‐dependent manner (Figure 5H). These results uncovered that ABHD17C is a BCL6B‐depalmitoylating enzyme.

To identify the ABHD17C‐mediated S‐palmitoylation sites on BCL6B, we obtained predicted palmitoylation sites from CSS‐Palm 4.0 and filtered them based on the palmitoylation score, which yielded five candidate cysteine residues: C59, C177, C389, C417, and C442 (Figure S5A). Among them, it should be noted that C442 is highly conserved across different species (Figure S5B). To determine the primary site of BCL6B palmitoylation, we mutated each cysteine to serine or all five cysteines to serines (5C‐5S) in BCL6B. The S‐palmitoylation levels were characterized using the azide palmitic acid incorporation assay. The mutations in five cysteine residues and the C442S mutation nearly eliminated the palmitoylation of BCL6B (Figure 5I), suggesting that Cys 442 is the major site for BCL6B palmitoylation. ABE assay confirmed that substitution of the Cys442 residue with serine by mutagenesis potently blocked the palmitoylation of exogenous BCL6B (Figure 5J). ABHD17C‐KO strikingly increased the palmitoylation of WT BCL6B in Mia paca‐2 cells, but not that of C442S mutant (Figure 5K). Consistent results were obtained in Panc‐1 cells with ABHD17C overexpression (Figure 5L). These results suggested that ABHD17C depalmitoylates BCL6B at cysteine 442. Given the effect of the ABHD17C/BCL6B axis on macrophage phagocytosis, we next investigated whether palmitoylation of Cys442 is indispensable for this process. For this purpose, we performed the phagocytosis assays of Mia paca‐2 and Panc‐1 cells expressing BCL6B WT or C442S mutant. Using fluorescence microscopy, we observed that pHRodo‐Red^+^ PC cells with C442S mutant were less susceptible to macrophage phagocytosis than the WT control (Figure 5M,N). In the context of the C442S mutation, ABHD17C‐KO or OE had no effect on the phagocytic capacity of macrophages (Figure 5M,N). Similarly, FCM analysis showed that PC cells harboring the C442S mutant exhibited a lower phagocytic percentage than the WT control (Figure 5O; Figure S4A). The BCL6B C442S mutation abrogated ABHD17C‐mediated regulation of macrophage phagocytosis (Figure 5O). Overall, these findings indicated that ABHD17C‐mediated depalmitoylation of BCL6B on Cys442 resists macrophage phagocytosis.

### 2.6 ABHD17C‐Mediated Depalmitoylation of BCL6B Promotes Its Degradation in a Ubiquitination Manner

**FIGURE 6:**
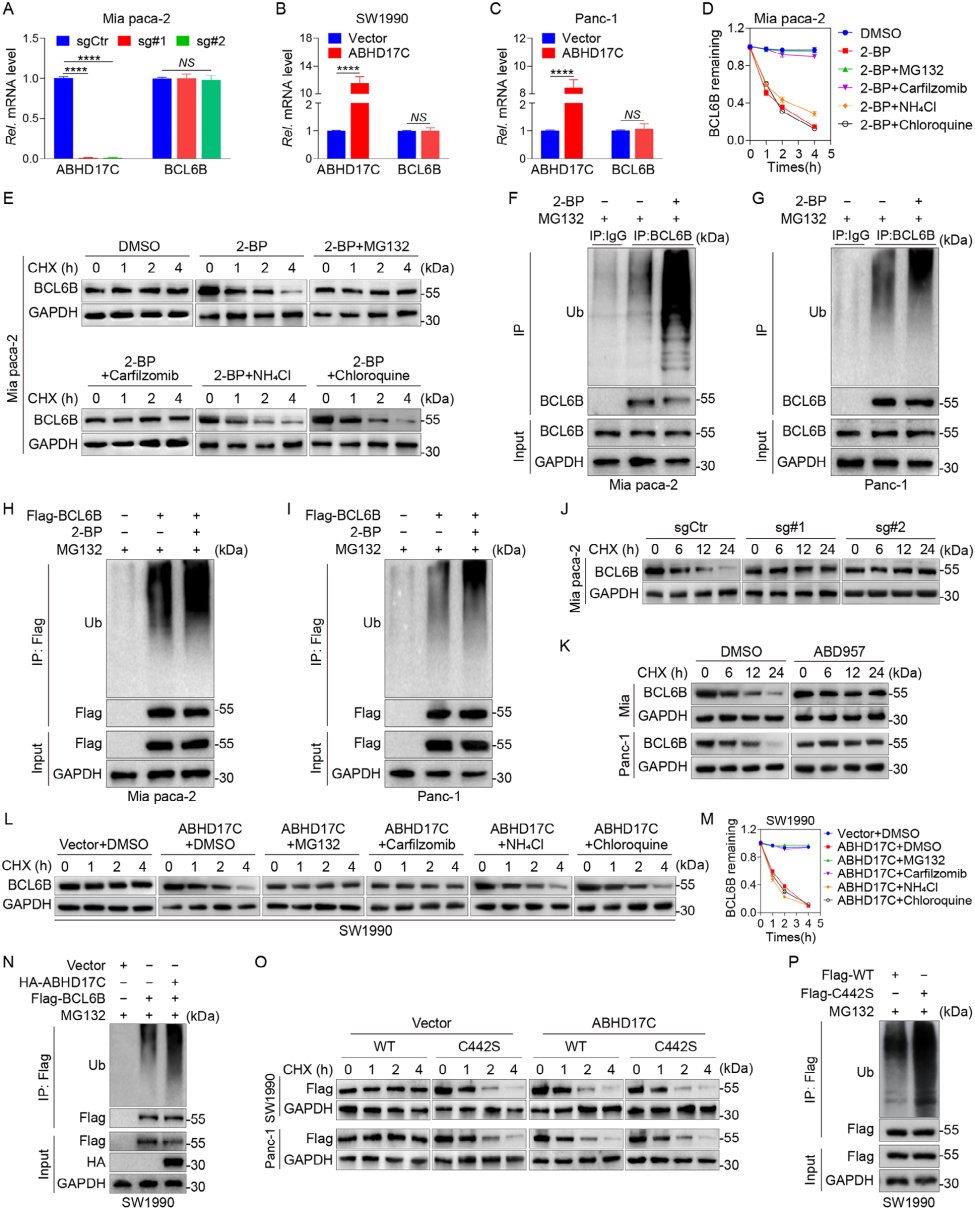
ABHD17C‐mediated depalmitoylation of BCL6B promotes its degradation in a ubiquitination manner. (A–C) qPCR quantification of ABHD17C and BCL6B mRNA in ABHD17C‐knockout Mia paca‐2 cells, and in ABHD17C‐overexpressing SW1990 and Panc‐1 cells. (D, E) The degradation of BCL6B in Mia paca‐2 cells treated with or without 2‐BP was detected by Cycloheximide (CHX) chase assay in the presence of proteasomal inhibitors (MG132 and Carfilzomib) and lysosomal inhibitors (NH_4_Cl and Chloroquine). Quantification of the intensity assessed by the relative level of BCL6B remaining. (F–I) Endogenous BCL6B or Flag‐tagged BCL6B ubiquitination levels were evaluated in Mia paca‐2 and Panc‐1 cells after treatment with or without 2‐BP, followed by MG132. (J, K) Upon ABHD17C knockout or treatment with the ABHD17 family‐specific inhibitor ABD957, BCL6B protein stability was assessed by CHX‐chase assay. (L, M) The degradation of BCL6B in SW1990 cells with or without ABHD17C overexpression was assessed by CHX‐chase assay in the presence of proteasomal inhibitors (MG132 and Carfilzomib) and lysosomal inhibitors (NH_4_Cl and Chloroquine). Quantification of the intensity is evaluated by the relative level of BCL6B remaining. (N) The ubiquitination levels of BCL6B were detected in SW1990 cells transfected with empty vector, or with Flag‐BCL6B, or with both HA‐ABHD17C and Flag‐BCL6B, followed by MG132 treatment. (O) The degradation of Flag‐BCL6B WT/C442S was analyzed by CHX‐chase assay in SW1990 and Panc‐1 cells with or without ABHD17C overexpression, following transfection with the Flag‐BCL6B WT/C442S plasmids. (P) The ubiquitination levels of BCL6B were detected in SW1990 cells transfected with Flag‐BCL6B WT/C442S plasmids, followed by MG132 treatment. Abbreviation: *Rel*., relative; DMSO, dimethyl sulfoxide; Ctr, control; 2‐BP, 2‐bromopalmitate; Ub, ubiquitin; IP, immunoprecipitation; CHX, cycloheximide; WT, wild‐type. Data are the mean ± SD of *n* = 3 (A–C, D, M) independent biological replicates; ^****^ *p* < 0.0001; *NS*, not significant. Statistical analysis was performed using a one‐way ANOVA with Dunnett's post‐hoc test (A), an unpaired two‐tailed Student's *t* test (B, C), or a two‐way repeated measures ANOVA with Bonferroni's post‐hoc test (D, M).

Given that palmitoylation is a reversible post‐translational modification known to regulate protein interactions and degradation [6], we therefore investigated whether ABHD17C regulates BCL6B stability and degradation via depalmitoylation. We found that knockout of ABHD17C increased only the protein level of BCL6B, but did not influence the transcriptional level of BCL6B in Mia paca‐2 cells (Figure 6A; Figure S4E). Consistent results were obtained in SW1990 and Panc‐1 cells with ABHD17C overexpression (Figure 6B,C; Figure S4E). 2‐BP was employed to depalmitoylate endogenous BCL6B in Mia paca‐2 and Panc‐1 cells treated with the protein synthesis inhibitor cycloheximide (CHX), and the effects of various inhibitors targeting different degradation pathways were subsequently assessed. 2‐BP treatment caused destabilization of BCL6B, which was rescued by the proteasome pathway inhibitors MG132 and Carfilzomib, but not lysosomal pathway inhibitors NH_4_Cl and Chloroquine (Figure 6D,E; Figure S5C,D). These results hinted that the proteasome pathway is the primary route for BCL6B degradation in PC cells. Upon inhibition of palmitoylation by 2‐BP, we found a robust increase in ubiquitinated degradation of endogenous and exogenous BCL6B when ubiquitin signals were accumulated using MG132 (Figure 6F–I), indicating that depalmitoylation modification destabilizes the BCL6B protein by promoting its ubiquitination. We then hypothesized that ABHD17C‐mediated depalmitoylation is responsible for this phenomenon observed with BCL6B. The results showed that knockout of ABHD17C, or treatment with the ABHD17C inhibitor ABD957, increased BCL6B stability (Figure 6J,K). ABHD17C overexpression induced BCL6B destabilization, which was likewise rescued by proteasome inhibitors MG132 and Carfilzomib, but not by lysosomal pathway inhibitors NH_4_Cl and Chloroquine (Figure 6L,M; Figure S5E,F). Furthermore, we observed increased ubiquitination of BCL6B upon ABHD17C overexpression, while decreased ubiquitination upon ABHD17C knockout (Figure 6N; Figure S5G). The substitution of the Cys442 residue with serine through mutagenesis accelerated the degradation of exogenous BCL6B in SW1990 and Panc‐1 cells treated with CHX (Figure 6O). The BCL6B WT exhibited instability similar to that of the BCL6B C442S in the ABHD17C‐OE cells (Figure 6O). The ubiquitination level of BCL6B C442S was significantly enhanced relative to BCL6B WT (Figure 6P; Figure S5H). Taken together, these data suggested that palmitoylation shields BCL6B from ubiquitination, whereas ABHD17C‐mediated BCL6B depalmitoylation drives its degradation in a ubiquitination manner.

### 2.7 ABHD17C‐Mediated BCL6B Depalmitoylation Inhibits Its Nuclear Translocation by Disrupting the Interaction With Importin‐α/β

**FIGURE 7:**
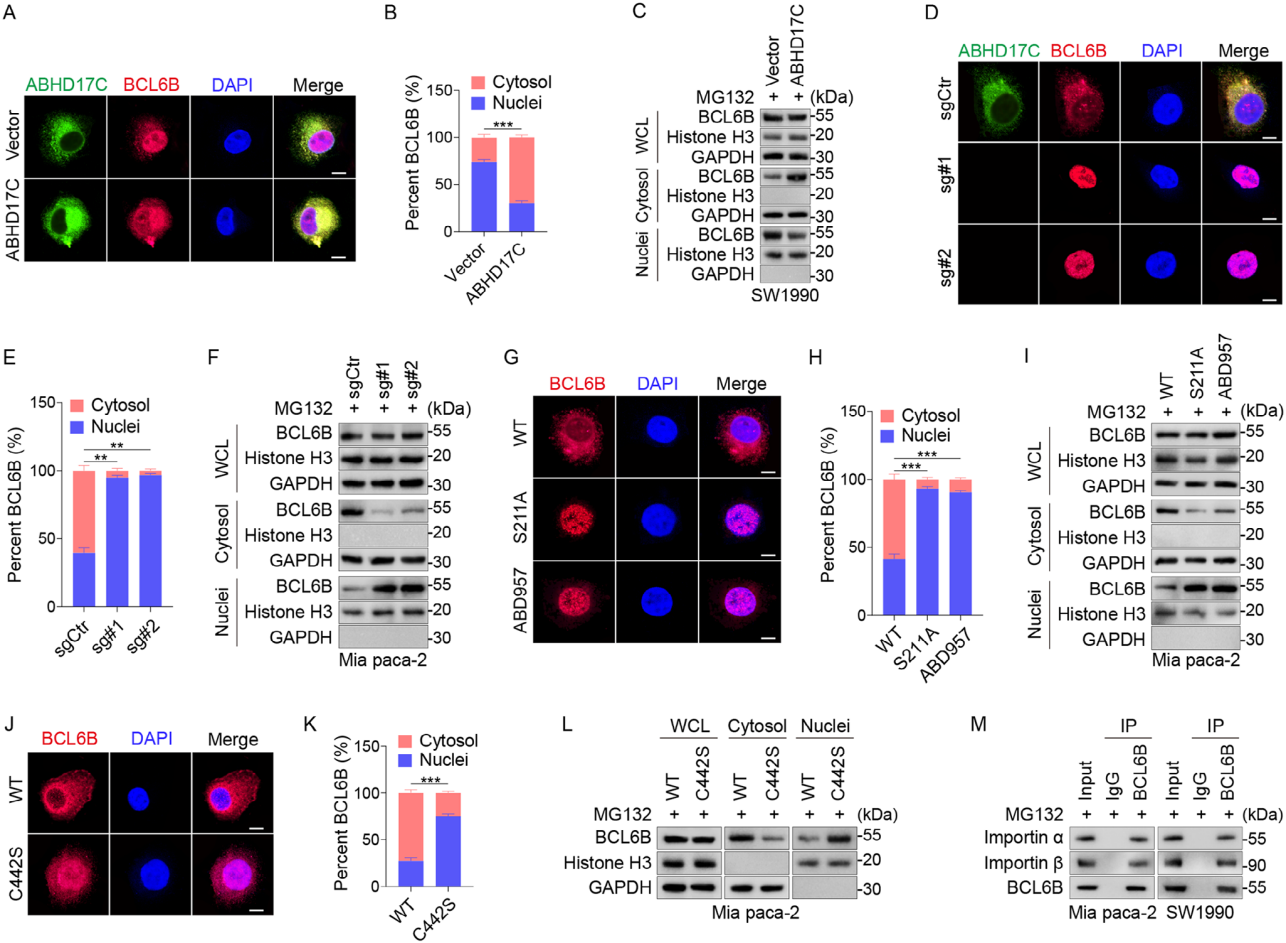
ABHD17C‐mediated BCL6B depalmitoylation inhibits its nuclear translocation by disrupting the interaction with importin‐α/β. (A) Representative immunofluorescence (IF) images of SW1990 cells infected with empty vector or ABHD17C plasmids, followed by MG132 treatment. Scale bar, 10 µm. (B) Statistical analysis of BCL6B fluorescence intensity in the nuclear and cytoplasmic compartments of the indicated groups. (C) Under MG132 treatment, Western blot analysis for cytosolic and nuclear fractions from SW1990 cells transfected with empty vector or ABHD17C plasmids. (D) Representative IF images of Mia paca‐2 cells with or without ABHD17C knockout, followed by MG132 treatment. Scale bar, 10 µm. (E) Statistical analysis of BCL6B fluorescence intensity in the nuclear and cytoplasmic compartments of the indicated groups. (F) Fractionation analysis of BCL6B in Mia paca‐2 cells with or without ABHD17C knockout, followed by MG132 treatment. (G) Representative IF images of Mia PaCa‐2 cells transfected with ABHD17C WT or S211A alone, or with ABHD17C WT combined with ABD957 treatment, followed by MG132 treatment. Scale bar, 10 µm. (H) Statistical analysis of BCL6B fluorescence intensity in the nuclear and cytoplasmic compartments of the indicated groups. (I) Under MG132 treatment, fractionation analysis of BCL6B in Mia paca‐2 cells transfected with ABHD17C WT or S211A alone, or with ABHD17C WT combined with ABD957 treatment. (J) Representative IF images of Mia paca‐2 cells transfected with BCL6B WT/C442S followed by MG132 treatment. Scale bar, 10 µm. (K) Statistical analysis of BCL6B fluorescence intensity in the nuclear and cytoplasmic compartments of the indicated groups. (L) Under MG132 treatment, fractionation analysis of BCL6B in Mia paca‐2 cells transfected with BCL6B WT/C442S. (M) Western blot analysis of endogenous importin α/β and BCL6B proteins following immunoprecipitation with anti‐BCL6B in Mia paca‐2 and SW1990 cells. Abbreviation: DAPI, 4’,6‐diamidino‐2‐phenylindole; WCL, whole cell lysate; Ctr, control; WT, wild‐type; IP, immunoprecipitation. Data are the mean ± SD of *n* = 3 (B, E, H, K) independent biological replicates; ^**^ *p* < 0.01, ^***^ *p* < 0.001. Statistical analysis was performed using an unpaired two‐tailed Student's *t* test (B, K) or a one‐way ANOVA with Dunnett's post‐hoc test (E, H).

Beyond regulating protein stability and degradation, palmitoylation modification is also crucially involved in protein trafficking and nuclear translocation [6, 23]. Given the role of ABHD17C in depalmitoylation, we sought to determine whether it influences the subcellular localization of BCL6B. To prevent ubiquitin‐mediated degradation, PC cells were pretreated with MG132. Immunofluorescence (IF) confocal microscopy for SW1990 and Panc‐1 cells showed that ABHD17C was localized exclusively in the cytoplasm, while BCL6B was distributed in both the cytoplasm and nucleus, with a predominant presence in the latter (Figure 7A; Figure S6A). Notably, co‐localization was observed between ABHD17C and BCL6B in the cytoplasmic compartment (Figure 7A; Figure S6A). ABHD17C overexpression induced a redistribution of BCL6B, characterized by a decrease in nuclear levels and a concomitant increase in cytoplasmic levels (Figure 7A; Figure S6A). Furthermore, it potentiated the co‐localization between ABHD17C and BCL6B within the cytoplasm (Figure 7A; Figure S6A). The quantitative fluorescence analysis is shown in Figure 7B and Figure S6B. To validate this observation, we performed subcellular fractionation to isolate cytosolic and nuclear fractions from cells. Consistently, the results confirmed that ABHD17C‐OE inhibited the nuclear transport of BCL6B (Figure 7C; Figure S6C). In contrast, knockout of ABHD17C shifted BCL6B localization from the cytoplasm to the nucleus (Figure 7D–F). To determine whether the blockade of nuclear import is dependent on ABHD17C‐depalmitoylating activity, the catalytically inactive S211A mutant and the selective depalmitoylase inhibitor ABD957 were employed. The results showed that S211A mutant and ABD957 strongly promoted BCL6B nuclear translocation in a palmitoylation‐dependent manner (Figure 7G–I; Figure S6D–F). More importantly, the C442S palmitoylation‐deficient mutation enhanced nuclear accumulation of BCL6B compared to WT control (Figure 7J–L; Figure S6G–I), indicating that BCL6B nuclear translocation is dependent on dynamic palmitoylation at Cys442. Importin‐α and importin‐β play crucial roles in nuclear protein import by forming ternary complexes with cytoplasmic protein [24]. Thus, we speculated that the nuclear translocation of BCL6B may be dependent on importin‐α/β. Expectedly, Co‐IP assays revealed that BCL6B directly bound to importin‐α/β (Figure 7M). Notably, Flag‐C442S mutant of BCL6B did not interact with importin‐α/β as Flag‐WT did (Figure S6J,K). Altogether, these findings suggested that ABHD17C‐mediated depalmitoylation of BCL6B at Cys442 inhibits its nuclear translocation by disrupting binding to importin‐α/β, thereby promoting BCL6B cytoplasmic retention and co‐localization with ABHD17C in PC cells.

### 2.8 BCL6B Directly Binds to the CD24 Promoter and Negatively Regulates Its Expression

**FIGURE 8:**
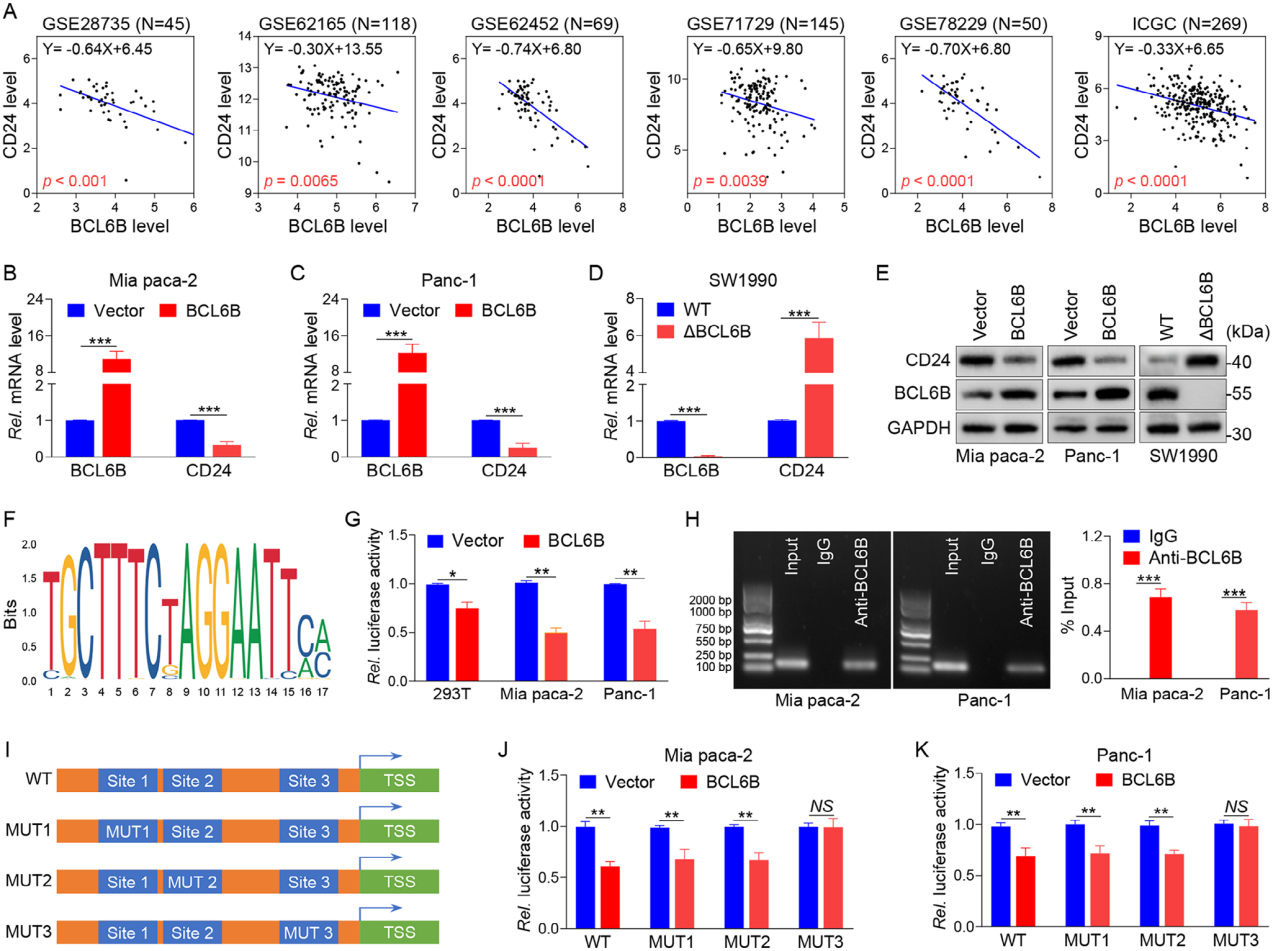
BCL6B binds directly to the CD24 promoter and negatively regulates its expression. (A) Analysis of the correlation between BCL6B and CD24 at the transcriptional level based on different public datasets, including five independent GEO datasets and the ICGC database. Sample sizes per dataset are indicated. (B–D) qPCR quantification of BCL6B and CD24 mRNA in BCL6B‐overexpressing Mia paca‐2 and Panc‐1 cells, and in BCL6B‐KO SW1990 cells. (E) The protein levels of BCL6B and CD24 were detected in BCL6B‐overexpressing Mia paca‐2 and Panc‐1 cells, as well as in BCL6B‐KO SW1990 cells. (F) The binding motif of BCL6B was predicted by the JASPAR database. (G) The effect of BCL6B on CD24 promoter activity was assessed using a dual‐luciferase reporter assay in 293T, Mia PaCa‐2, and Panc‐1 cells. (H) The binding of transcriptional repressor BCL6B to the promoter of CD24 in Mia paca‐2 and Panc‐1 cells was determined by chromatin immunoprecipitation (ChIP) assay. Anti‐BCL6B antibody enrichment at the CD24 locus was assessed by ChIP‐PCR. (I) Schematic diagram of TSS, MUT1, MUT2, and MUT3 mutation site locations. (J, K) The regulatory interplay between BCL6B protein and the CD24 promoter at the predicted binding sites was analyzed in Mia paca‐2 and Panc‐1 cells using a dual‐luciferase reporter assay. Abbreviation: *Rel*., relative; WT, wild‐type; MFI, mean fluorescence intensity; MUT, mutation site; TSS, transcription start site. Data are the mean ± SD of *n* = 3 (B–D, G, H, J, K) independent biological replicates; ^*^ *p* < 0.05, ^**^ *p* < 0.01, ^***^ *p* < 0.001; *NS*, not significant. Statistical analysis was performed using two‐tailed Spearman's test (A) or an unpaired two‐tailed Student's t test (B–D, G, H, J, K).

Given the documented function of BCL6B as a key transcriptional repressor [25], we postulated CD24 to be a critical downstream target. Integrated analysis of data from five Gene Expression Omnibus (GEO) datasets (GSE28735, GSE62165, GSE62452, GSE71729, and GSE78229) and the International Cancer Genome Consortium (ICGC) database revealed a consistent inverse correlation between BCL6B and CD24 at the transcriptional level (Figure 8A). Overexpression of BCL6B significantly reduced the expression of CD24 in both mRNA and protein levels in Mia paca‐2 and Panc‐1 cells, while knockout of BCL6B (ΔBCL6B) dramatically increased the expression of CD24 in both mRNA and protein levels in SW1990 cells (Figure 8B–E; Figure S7A,B). Therefore, it is likely that the regulation occurs at the transcriptional level. The binding motif of BCL6B was obtained from JASPAR (Figure 8F). Next, potential BCL6B binding sites within the CD24 promoter region was predicted using the open‐access database JASPAR (Figure S7C). To confirm the suppression of CD24 expression was directly mediated by BCL6B, we cloned CD24 promoter regions containing predicted transcription factor binding sites (TFBS) into the pGL3‐Basic reporter vector. Luciferase reporter assays revealed that BCL6B inhibited the promoter activities of CD24 in 293T, Mia paca‐2 and Panc‐1 cells (Figure 8G). Chromatin immunoprecipitation (ChIP) assay showed that CD24 enrichment was increased in the anti‐BCL6B groups (Figure 8H). To validate the three predicted binding sites from JASPAR, we performed site‐directed mutagenesis (Figure 8I; Figure S7D). Luciferase reporter assays demonstrated that the transcriptional inhibition was specific to binding site 3 (MUT3), as mutation of binding site 1 (MUT1) or site 2 (MUT2) did not abrogate repression (Figure 8J,K). Collectively, these data substantiated that BCL6B acts as a transcriptional repressor by directly binding to the CD24 promoter to suppress its expression.

### 2.9 Loss of BCL6B Protects PC Cells From Macrophage Attack by Enhancing CD24/Siglec‐10 Signaling

**FIGURE 9:**
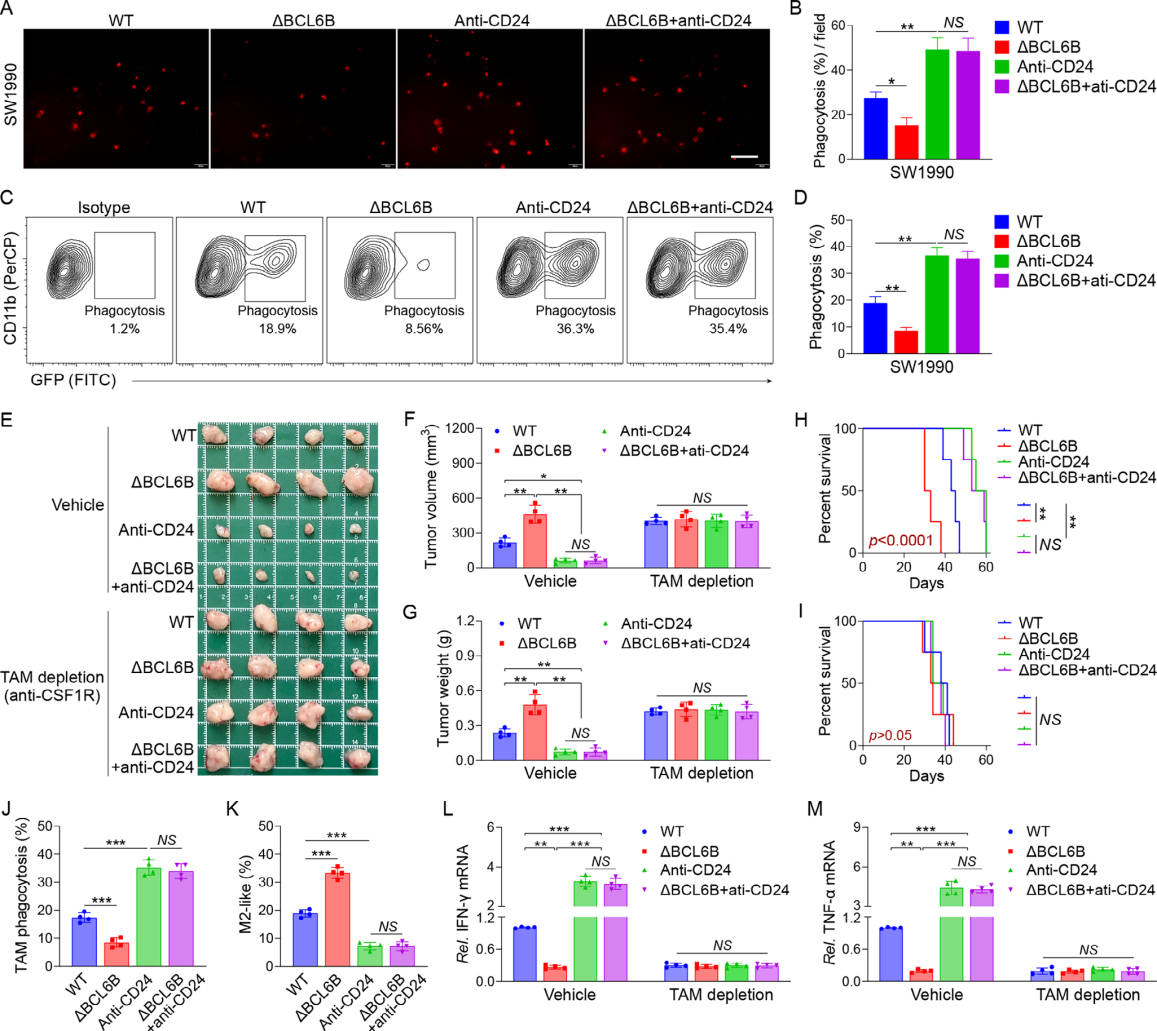
Loss of BCL6B protects PC cells from macrophage attack by enhancing CD24‐Siglec‐10 signaling. (A, B) Representative images of phagocytosis assays. Peripheral blood mononuclear cell (PBMC)‐derived macrophages were co‐cultured with pHrodo‐red^+^ SW1990 cells with or with BCL6B‐KO, and pre‐incubated with IgG control or anti‐CD24 mAb. Red puncta inside macrophages indicate engulfed SW1990 cells. Scale bar, 100 µm. (C, D) Representative flow cytometry (FCM) plots showing phagocytosis of GFP^+^ SW1990 cells with or with BCL6B knockout, and pre‐treated with IgG control or anti‐CD24 mAb. CD11b^+^ GFP^+^ cells were defined as engulfed cells. (E) Representative images of orthotopic pancreatic cell‐derived xenografts (CDXs) in NSG mice. *n* = 5 mice per group. Burden of SW1990 WT/ΔBCL6B tumors treated with or without anti‐CD24 mAb in mice with tumor‐associated macrophages (TAMs) (vehicle) or TAM‐depleted mice (anti‐CSF1R) was evaluated. Tumors were excised, photographed, and weighed at day 28. (F, G) Excised tumor volume (mm^3^) and mass (g) were measured. (H, I) Kaplan–Meier survival analysis of an independent NSG mouse cohort following orthotopic inoculation in the indicated treatment groups. *n* = 5 mice per group. (J) The frequency of phagocytosis events out of all TAMs within tumor masses among different groups. (K) The relative abundance of CD206^+^ (M2‐like) TAMs among all TAMs was determined. (L, M) The mRNA level of TNF‐α and IFN‐γ was detected in tumor tissues with or with TAM depletion across different groups. Abbreviation: WT, wild‐type; TAM, tumor‐associated macrophage; *Rel*., relative. Data are the mean ± SD of *n* = 3 (B, D) and *n* = 4 (F–M) independent biological replicates; ^*^ *p* < 0.05, ^**^ *p* < 0.01, ^***^ *p* < 0.001; *NS*, not significant. Statistical analysis was performed using a one‐way ANOVA with Tukey's post‐hoc test (B, D, J, K) or a two‐way ANOVA with Tukey's post‐hoc test (F, G, L, M), and survival curves were compared using the Log‐rank (Mantel‐Cox) test (H, I).

Next, we explored whether BCL6B influences macrophage phagocytosis by modulating “don't eat me” signal CD24. Under the fluorescence microscope, it was observed that that ΔBCL6B‐pHRodo‐Red^+^ cells were harder engulfed and degraded in the low‐pH phagolysosome as compared to WT cells, but these treated with a CD24 blocking mAb were more readily engulfed and rescued the phagocytosis‐inhibiting effect of ΔBCL6B (Figure 9A,B). Similarly, the FCM‐based measurements of phagocytosis showed that, compared with that of WT cells, the phagocytosis of these PC cells with ΔBCL6B by macrophages was decreased, while blockade of CD24 increased its phagocytosis and abolished the effect of ΔBCL6B (Figure 9C,D). Furthermore, blocking Siglec‐10 significantly enhanced macrophage phagocytosis and eliminated the ΔBCL6B‐mediated phagocytosis resistance (Figure S7E,F). These results indicated that BCL6B relies on CD24/Siglec‐10 axis to regulate macrophage phagocytosis.

To investigate whether the phagocytosis inhibition conferred by BCL6B deletion could be recapitulated in vivo, an in situ pancreatic CDX model was established in NSG mice (Figure S7G). Several weeks post‐engraftment, we found that loss of BCL6B significantly accelerated the growth of the tumor as compared to WT control (Figure 9E–G). CD24 blockade suppressed tumor growth and abolished the pro‐tumoral role mediated by ΔBCL6B (Figure 9E–G), hinting that BCL6B relies on the anti‐phagocytic factor CD24 to influence the malignant progression of PC. Notably, TAM depletion not only abolished the pro‐tumoral role of ΔBCL6B but also eliminated the anti‐tumoral effect of anti‐CD24 mAbs (Figure 9E–G; Figure S7H), indicating that the alteration in tumor burden induced by BCL6B or CD24 is dependent on TAM‐mediated clearance. This growth difference, attributable to impaired phagocytic clearance, significantly shortened the survival time of mice engrafted with BCL6B‐deficient tumors, while CD24 blockade prolonged survival of mice, reversed the ΔBCL6B‐mediated survival disadvantage and improved outcome (Figure 9H). TAM depletion abolished the survival‐altering effects mediated by ΔBCL6B and CD24 blockade (Figure 9I). FCM‐based analysis confirmed that knockout of BCL6B reduced TAM phagocytosis, while CD24 blockade enhanced TAM‐mediated clearance, and abrogated ΔBCL6B‐mediated phagocytosis resistance in vivo (Figure 9J). As expected, ΔBCL6B increased the abundance of M2‐like TAMs within the tumor niche, while CD24 blockade reversed the pro‐M2 effect induced by BCL6B deletion (Figure 9K). ΔBCL6B tumors exhibited significantly reduced levels of the anti‐tumoral cytokines TNF‐α and IFN‐γ; in contrast, CD24 blockade boosted the levels of TNF‐α and IFN‐γ, and abrogated the inhibitory effect of ΔBCL6B on these cytokines (Figure 9L,M). Besides, TAM depletion eliminated differences in TNF‐α and IFN‐γ levels within the tumor microenvironment across all groups (Figure 9L,M). Altogether, these finding suggested that BCL6B loss protects PC cells from macrophage attack via the anti‐phagocytic signal CD24, fosters an immunosuppressive microenvironment, and promotes PC progression in vivo.

### 2.10 ABHD17C‐Mediated Depalmitoylation of BCL6B‐Cys442 Enhances CD24 Transcription

**FIGURE 10:**
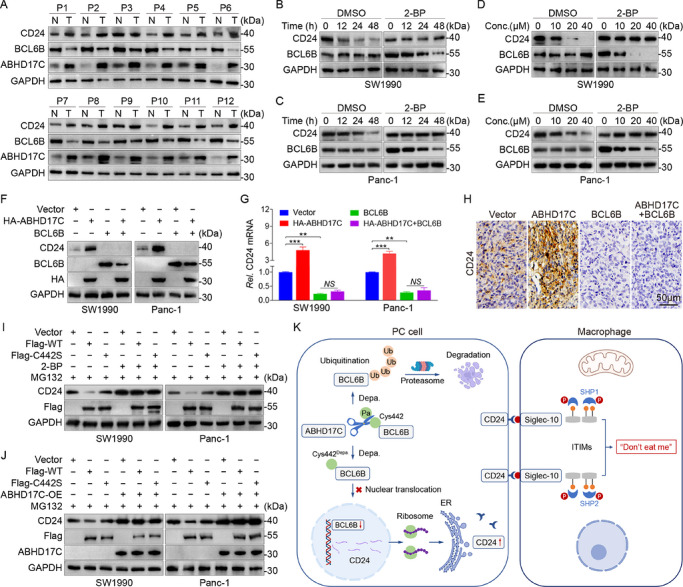
ABHD17C‐mediated depalmitoylation of BCL6B‐Cys442 enhances CD24 transcription. (A) Western blot analysis for the expression of ABHD17C/BCL6B/CD24 axis in 12 paired pancreatic cancer (PC) tissues and adjacent normal tissues (ANTs). (B, C) Western blot analysis for the expression of BCL6B and CD24 in SW1990 and Panc‐1 cells with DMSO/2‐BP treatment for 0, 12, 24, and 48 h. (D, E) Western blot analysis for the expression of BCL6B and CD24 in SW1990 and Panc‐1 cells with DMSO/2‐BP treatment for 0, 10, 20, 40 µm. (F) Western blot analysis for the expression of HA, BCL6B, and CD24 in SW1990 and Panc‐1 cells transfected with HA‐ABHD17C plasmids, with or without BCL6B co‐transfection. (G) qPCR analysis for the mRNA level of CD24 in SW1990 and Panc‐1 cells transfected with HA‐ABHD17C plasmids, with or without BCL6B co‐transfection. (H) Representative immunohistochemistry (IHC) staining images for CD24 in tumor masses of Figure 4K. Orthotopic pancreatic cell‐derived xenografts (CDXs) engrafted with Panc‐1 cells transfected with ABHD17C or BCL6B, or co‐transfected with both. Scale bar, 50 µm. (I) Under MG132 treatment, Western blot analysis for the expression of Flag‐BCL6B and CD24 in SW1990 and Panc‐1 cells infected with vector, Flag‐BCL6B WT/C442S plasmids, with or without 2‐BP treatment. (J) Under MG132 treatment, Western blot analysis for the expression of Flag‐BCL6B and CD24 in SW1990 and Panc‐1 cells infected with vector, Flag‐BCL6B WT/C442S plasmids, with or without ABHD17C overexpression. (K) Schematic diagram of PC cells resisting macrophage phagocytosis via the ABHD17C/BCL6B/CD24 depalmitoylation axis. Abbreviation: P, patient; N, normal; T, tumor; DMSO, dimethyl sulfoxide; Conc., concentration; *Rel*., relative; WT, wild‐type; 2‐BP, 2‐bromopalmitate; OE, overexpression. PC, pancreatic cancer; Ub, Ubiquitination; Pa, palmitoylation; Depa., depalmitoylation; Cys, cysteine; ER, endoplasmic reticulum; SHP, Src homology 2 domain‐containing protein tyrosine phosphatase; ITIMs, immunoreceptor tyrosine‐based inhibitory motifs. Data are the mean ± SD of *n* = 3 (G) independent biological replicates; ^**^ *p* < 0.01, ^***^ *p* < 0.001; *NS*, not significant. Statistical analysis was performed using a one‐way ANOVA with Tukey's post‐hoc test (G).

Next, we examined whether ABHD17C promotes CD24 expression by destabilizing BCL6B via depalmitoylation, thereby alleviating its transcriptional inhibition. The protein levels of the ABHD17C/BCL6B/CD24 axis were evaluated in 12 paired PC and ANT tissues, revealing a reciprocal relationship characterized by high expression of ABHD17C and CD24 but low expression of BCL6B (Figure 10A). Multiplex IF staining of PC tissues further confirmed the co‐localization between ABHD17C and BCL6B, and the reciprocal expression pattern of ABHD17C, BCL6B and CD24 (Figure S8A). Furthermore, treatment with 2‐BP potently suppressed CD24 expression in both a time‐dependent and dose‐dependent manner (Figure 10B–E). At both the protein and mRNA levels, ectopic ABHD17C expression upregulated CD24 expression, an effect that was blocked by BCL6B (Figure 10F,G). IHC results from tumor tissue sections of NSG confirmed that ABHD17C elevated CD24 expression compared to vector control, whereas BCL6B not only reduced baseline CD24 levels but also completely reversed the ABHD17C‐driven increase in vivo (Figure 10H; Figure 4K). In ABHD17C‐KO or BCL6B‐OE cells, restoration of ABHD17C‐WT effectively destabilized BCL6B, reduced BCL6B expression, increased CD24 levels (Figure S8B–D), and suppressed macrophage phagocytosis (Figure S8E–H). In contrast, the catalytically inactive mutant ABHD17C‐S211A failed to reverse these alterations (Figure S8B–H). Moreover, the BCL6B Cys442 mutation did not decrease CD24 expression as BCL6B WT did (Figure 10I,J). However, after palmitoylation blockade by 2‐BP treatment or ABHD17C‐OE, there was no significant difference in CD24 expression between the C442S and WT groups (Figure 10I,J). As shown above (Figure 4I–M), BCL6B overexpression reversed the promotive effect of ectopic ABHD17C expression on PC both in vitro and in vivo. Taken together, these results suggested that ABHD17C‐mediated depalmitoylation of BCL6B at Cys442 enhances CD24 transcription to resist macrophage phagocytosis and to promote PC progression.

## 3 Discussion

Published studies have established that palmitoylated proteins have significant clinical relevance in multiple tumor types [26, 27, 28]. In hepatocellular carcinoma, highly expressed ZDHHC20 is negatively associated with clinical outcomes of patients [27]. In glioma, ZDHHC17 abundance increases incrementally from normal brain tissue through grade I‐IV specimens [28]. In this study, we leveraged multiple public databases and a TMA‐IHC cohort to clarify the clinical significance of the depalmitoylase ABHD17C in PC. The data show that ABHD17C is significantly upregulated in PC tissues and predicts shorter OS and DFS. Multivariate analysis further identifies ABHD17C as an independent risk factor for PC survival. Moreover, its expression level positively correlates with larger tumor size, lymph‐node metastasis, and higher TNM stage. Collectively, ABHD17C may serve as a promising prognostic indicator for patients with PC.

More than twenty palmitoyltransferases have been identified, with the ZDHHC family being particularly prominent [7]. In contrast, only a limited number of depalmitoylases are known, such as APT1, APT2, PPT1, PPT2, and the recently described ABHD17 family [26]. The substrate spectra and enzymatic specificities of ABHD17 family remain largely uncharacterized. Therefore, the precise molecular mechanisms through which depalmitoylation drives PC progression have not yet been fully characterized. Here, we interrogated ABHD17C, a depalmitoylase first characterized in 2015 for its role in N‐Ras palmitoylation cycling [8], and delineated its previously unrecognized capacity to depalmitoylate BCL6B at Cys442, a modification that critically regulates the protein stability of BCL6B and influences PC progression.

Recent years have witnessed substantial progress in elucidating the roles of protein S‐palmitoylation in tumor initiation and progression through multiple mechanisms, encompassing invasion, metastasis, stem‐cell‐like properties, drug resistance, and the regulation of pivotal oncogenic signaling pathways [26, 28, 29, 30]. Few studies have yet reported how palmitoylation reshapes the tumor immune microenvironment and accelerates malignant progression. ZDHHC3‐mediated palmitoylation of PD‐L1 has been shown to inhibit its degradation, thereby stabilizing the protein and potentiating T‐cell cytotoxicity suppression [31]. In the present study, we focused on the innate immune checkpoint governing macrophage phagocytosis, rather than T cell‐mediated immune killing. Our data demonstrated that ABHD17C‐catalyzed depalmitoylation of BCL6B dictates its nuclear translocation and protein stability, which in turn tunes expression of anti‐phagocytic signal CD24, and ultimately controls TAM phagocytosis within the TME of PC. These findings uncover a new mechanism mediated by depalmitoyltransferase ABHD17C that plays a critical role in innate immune phagocytosis.

Previous reports have indicated that palmitoylation modification affects the ubiquitin‐proteasome degradation process [31]. PD‐L1 undergoes palmitoylation within its cytoplasmic tail; this lipid modification shields the protein from ubiquitination, thereby preventing lysosomal degradation and prolonging its half‐life [31]. FASN palmitoylation catalyzed by ZDHHC20 competed against the ubiquitin‐proteasome pathway via the E3 ubiquitin ligase complex [27]. Through co‐IP, PLA assays, and fluorescence confocal microscopy, we observed the interaction between ABHD17C and BCL6B as well as their co‐localization in the cytoplasm of PC cells. Furthermore, ABHD17C‐mediated depalmitoylation of BCL6B at Cys442 accelerates its degradation via the ubiquitin‐proteasome pathway, rather than the lysosomal pathway. This inverse regulatory relationship is also evident at the clinical observations. Intriguingly, high expression of ABHD17C and low expression of BCL6B are observed in PC tissues. Elevated ABHD17C predicts poor clinical outcomes, whereas increased BCL6B is associated with favorable prognosis in patients with PC. Of note, the mechanism by which palmitoylation influences protein ubiquitination and degradation remain to be further elucidated. We speculate that depalmitoylation may increase the probability of recognition by the ubiquitin‐proteasome system, possibly by physically exposing ubiquitination sites or degradation‐related domains on the protein surface.

Beyond protein stability, palmitoylation also serves as a trafficking code that regulates their precise membrane microdomains and organellar localization [23, 26, 32]. ZDHHC1‐mediated palmitoylation of p53 at Cys135, Cys176, and Cys275 facilitates its nuclear translocation, supporting the tumor‐suppressive function of p53 [23]. Under physiological conditions, BCL6B interacts with BCL6 via BTB/POZ domain, accumulates in the nucleus, and occupies BCL6‐specific DNA elements to act as a transcriptional repressor [33]. Herein, our data reveal that BCL6B depalmitoylation at Cys442 traps the transcription factor in the cytoplasm, preventing its nuclear entry by disrupting the interaction with importin‐α/β; the resulting drop in nuclear BCL6B lifts its repression of CD24 and amplifies the “don't eat me” signal in PC. The precise molecular mechanism by which BCL6B depalmitoylation inhibits nuclear translocation remains to be fully elucidated; we postulate that increased hydrophilicity or conformational changes following depalmitoylation may impair its interaction with nuclear transport proteins.

BCL6B is emerging as a context‐dependent tumor suppressor whose transcriptional repressor activity underpins multiple anti‐oncogenic programs [33]. It halts PI3K/AKT signaling to curb colorectal cancer cell proliferation and migration [34], and its promoter hyper‐methylation frequented in gastric cancer predicts shortened survival [35]. Nevertheless, the function of BCL6B in PC remains unknown. Herein, we uncover that depalmitoylation of BCL6B redirects it to cytoplasmic ubiquitination and degradation, thereby reducing nuclear BCL6B levels and decreased binding of BCL6B to the CD24 promoter region in PC cells. This relieves BCL6B‐mediated transcriptional repression of CD24, leading to upregulated expression of the anti‐phagocytic signal CD24 and ultimately enabling escape from macrophage‐mediated engulfment in vivo. Our findings suggest that mapping the newly identified regulators of BCL6B expression may expose novel drivers of PC pathogenesis.

Recent studies have identified the CD24/Siglec‐10 axis as a critical “don't eat me” signal in tumor immunity [18, 36, 37, 38], including ovarian cancer, breast cancer and lung cancer. This pathway functions as a potent innate immune checkpoint; therapeutic anti‐CD24 antibodies bind tumor‐expressed CD24, disrupt the CD24/Siglec‐10 interaction, and thereby resist macrophage‐mediated phagocytosis [37]. CD24 expression on tumor cells is governed by a diverse array of regulatory factors [36, 37, 38]. Mesothelin upregulates CD24 level via Wnt/β‐catenin signaling, thereby fostering a pro‐tumorigenic phenotype in TAMs [36]. Herein, we uncover a novel mechanism driving CD24 upregulation to escape macrophage‐mediated innate immune surveillance. Loss of the transcriptional repressor BCL6B enhances transcription of the “don't eat me” signal CD24, protecting PC cells from macrophage attack. Anti‐CD24 mAb or anti‐Siglec‐10 mAb abrogates the ability of ABHD17C or BCL6B to modulate macrophage‐mediated clearance, indicating that the immunoregulatory effects of ABHD17C and BCL6B are strictly dependent on the CD24/Siglec‐10 anti‐phagocytic signaling. This notion was further corroborated by the observation that macrophage depletion in NSG mice eliminated the effects mediated by BCL6B and anti‐CD24 mAb. Although orthotopic PC‐CDX models in NSG mice effectively investigate the interactions between macrophages and PC cells in vivo, the model lacks adaptive immunity. This deficiency compromises a comprehensive assessment of the tumor immune microenvironment, and future studies in immunocompetent models are needed to validate these findings. Moreover, blockade with anti‐CD24 mAb induced macrophage polarization, consistent with previous reports in other tumors [18, 36]. It is possible that the altered phagocytic capacity of macrophages is related to their polarization. Collectively, elucidating the dynamic balance ABHD17C‐mediated BCL6B depalmitoylation and BCL6B‐mediated transcriptional silencing of CD24 could lay the groundwork for therapeutic strategies to disrupt the macrophage phagocytosis signaling and improve clinical outcomes for patients with PC.

Therapeutically, the ABHD17C/BCL6B/CD24 axis presents multiple intervention opportunities. While clinical‐grade selective ABHD17C inhibitors remain to be developed beyond the research tool ABD957, CD24‐targeted therapies have already advanced into Phase I trials with first‐in‐class antibodies such as ATG‐031 [39], supporting the translational relevance of disrupting this “don't eat me” signal. Notably, stabilizing nuclear BCL6B through small molecules that inhibit its depalmitoylation or ubiquitination represents a promising yet unexplored strategy to restore transcriptional repression of CD24 and potentiate macrophage‐mediated antitumor immunity.

In conclusion, ABHD17C is significantly elevated in PC and correlated with unfavorable clinical outcomes. By catalyzing the depalmitoylation of BCL6B at Cys442, ABHD17C blocks the importin‐α/β‐mediated nuclear translocation of BCL6B and redirects it toward ubiquitin‐proteasome degradation. Loss of nuclear BCL6B alleviates transcriptional repression of the “don't eat me” signal CD24, increases its expression, enables PC cells to evade engulfment by TAMs within TME and ultimately accelerating PC progression (schematic in Figure 10K). These findings underscore the potential of targeting the ABHD17C/BCL6B/CD24 signaling axis as a therapeutic strategy for PC.

## 4 Materials and Methods

### 4.1 Data Mining and Bioinformatics Analysis

Multiple publicly accessible databases were employed to investigate ABHD17C expression, evaluate its prognostic significance, and conduct associated analyses, including GEPIA [40], TNMplot [41], The Human Protein Atlas [42], LinkedOmics [43], TISIDB [44], Kapla‐Meier plotter [45], TIMER [46], and GEO (https://www.ncbi.nlm.nih.gov/gds). In GEPIA, ABHD17C expression was analyzed utilizing the “Match TCGA normal and GTEx data” option; the cutoff setting “Cutoff‐High versus Cutoff‐Low: 42 versus 42” was selected for survival analysis. Unpaired datasets GSE16515 and GSE62165 and paired datasets GSE15471 and GSE28735 were used to compare the transcriptional level of ABHD17C between PC tissues and normal pancreatic tissues. The GSE71729 dataset was analyzed to explore the transcriptional level of ABHD17C across normal pancreatic tissue, in situ PC tissue, and liver and lung metastasis foci. The transcriptional correlation between BCL6B and CD24 was examined using five GEO datasets (GSE28735, GSE62165, GSE62452, GSE71729, and GSE78229) and the ICGC database [47]. All GEO datasets were processed using quantile normalization to enable direct comparisons across the selected samples. The single‐cell RNA sequencing data (CRA001160) from Tumor Immune Single‐Cell Hub (TISCH) was used to explore the differential expression patterns of ABHD17C across various cellular compartments in PC tissue, including cancer cells, normal ductal cells, fibroblasts, and immune cell populations [48]. The CSS‐Palm 4.0 algorithm was applied to identify potential palmitoylation sites on the BCL6B protein [49]. The open‐access JASPAR database was interrogated to predict potential BCL6B TFBS in the CD24 promoter region [50].

### 4.2 Patients and Samples

A commercial PCTMA containing 97 PC tissues and 89 ANTs was obtained from Shanghai Outdo Biotech Co., Ltd. Among them, 8 paired ANT samples were missing. The specimens were collected between January 2014 and October 2015. Inclusion criteria: (1) age ≥ 18 years; (2) pathologically confirmed pancreatic ductal adenocarcinoma; (3) underwent pancreatoduodenectomy or distal pancreatectomy. Exclusion criteria: (1) neoadjuvant radiotherapy or chemotherapy; (2) death within 3 months of surgery; (3) refused follow‐up. Staging was assigned according to the eighth edition of the American Joint Committee on Cancer manual. Median age was 67 years (range 42–78). Patients were followed every 3–6 months; the longest follow‐up was 114 months. At the last census, 70 patients had died and 27 remained alive. Complete clinicopathological and follow‐up data were available for all 97 cases (Table S1). Additionally, 12 pairs of freshly resected PC and matched ANT samples were snap‐frozen in liquid nitrogen and stored at −80°C for western blotting of the ABHD17C/BCL6B/CD24 axis. Human peripheral blood was obtained from three healthy donors (two males, one female; average age 30.7 years). The study was approved by the Ethics Committee of the First Affiliated Hospital of USTC (No. 2024‐KY‐246). All participants provided written informed consent before enrollment.

### 4.3 Hematoxylin and Eosin (H&E), and Immunohistochemical (IHC) Staining

H&E and IHC staining were performed according to the standard procedures previously described [51, 52]. The primary antibodies used for IHC staining are listed below: anti‐ABHD17C antibody (1:200, ab151040, Abcam), anti‐F4/80 antibody (1:200, ab111101, Abcam), anti‐CD68 antibody (1:200, ab201340, Abcam), and anti‐CD24 antibody (1:200, sc‐19585, Santa Cruz Biotechnology). TMA‐IHC assessment of ABHD17C was performed independently by two senior pathologists who were blinded to the follow‐up data of PC patients. For any given section with divergent scores, a final consensus was reached after joint re‐examination. IHC scores were determined based on staining intensity (graded as 0, 1, 2, or 3) and the percentage of positive cells (ranging from 0% to 100%). The final IHC score was derived by multiplying the intensity score by the positivity rate [53, 54]. Using the median IHC score as the cutoff, the samples were categorized into low‐ and high‐ABHD17C expression groups.

### 4.4 Cell Lines and Culture

The human immortalized pancreatic ductal epithelial cell line (HPNE), PC cell lines (Panc‐1, AsPC‐1, BxPC‐3, Mia paca‐2, SW1990, CFPAC‐1, and T3M4), and HEK293T were purchased from the American Type Culture Collection (ATCC, USA) or National Collection of Authenticated Cell Cultures (Shanghai, China). Cells were cultured in DMEM, RPMI‐1640, or L‐15 medium (Gibco) supplemented with 10% fetal bovine serum (FBS; Gibco) and 1% Penicillin–Streptomycin (Gibco) at 37°C with 5% CO_2_. The culture medium for HPNE cells was supplemented with recombinant human epidermal growth factor (P00033, Solarbio) at a final concentration of 10 ng/mL. The cell‐culture supernatants were routinely tested for Mycoplasma to ensure the absence of contamination.

### 4.5 Reagents

Dimethyl sulfoxide (DMSO; D8371, Sorlabio), palmitoylation inhibitor 2‐BP (50 µm for 24 h; 21604, Sigma–Aldrich), ABHD17C inhibitor ABD957 (500 nm or indicated concentration for 6 h; HY‐142161, Med Chem Express); protein synthesis inhibitor CHX (100 µg/mL at indicated timepoints; HY‐12320, Med Chem Express), proteasome inhibitor MG132 (10 µm for 6 h; M8699, Sigma–Aldrich), proteasome inhibitor Carfilzomib (50 nm for 6 h; S2853, Selleck), lysosome inhibitor NH_4_Cl (20 mm for 6 h; HY‐Y1269, Med Chem Express), and lysosome inhibitor Chloroquine (50 µm for 6 h; HY‐17589A, Med Chem Express).

### 4.6 Lentiviral Infection and CRISPR‐Mediated Gene Knockout (KO)

Parental Mia paca‐2 and SW1990 were infected with GFP‐puromycin lentivirus (Genechem) to generate Mia paca‐2‐GFP‐Puro^+^ and SW1990‐GFP‐Puro^+^ cell lines using Lipofectamine 2000 Reagent (Thermo Fisher Scientific, USA) according to the manufacturer's instructions. After 48 h, cells were harvested and isolated by fluorescence‐activated cell sorting to obtain pure populations. The ABHD17C‐KO of Mia paca‐2 and Panc‐1 cells were generated by electroporating cells with recombinant CRISPR/Cas9 ribonucleoprotein (RNP). CRISPR/Cas9 sgRNAs targeting human ABHD17C/BCL6B assembled with Cas9‐3NLS nuclease via incubation at 37°C for 60 min. A total of 2 × 10^5^ Mia paca‐2‐GFP^+^ or SW1990‐GFP^+^ cells were harvested, mixed with relative Cas9/RNP complexes, and electroporated using the Neon Transfection System (MPK5000, Invitrogen) with two pulses of 1150 V, 30 ms. After 48 h of recovery, genetically‐modified cells were selected with 2 µg/mL concentration of puromycin (ST551, Beyotime) for 7 days to establish stable integration. The sgRNA sequences for CRISPR/Cas9 mediated‐gene KO are as follows: *hABHD17C* sgRNA#1: CGAGCCCACCTACACGGTGC, *hABHD17C* sgRNA#2: ACCTCAGCGAGCGCGCCGAC, and *hBCL6B* sgRNA: TCGCCACTCCTCCGACGTGCTGG.

### 4.7 Plasmid Construction

Lentiviral plasmids were constructed by Shanghai Genechem Co., Ltd. The coding sequences of human ABHD17C and BCL6B were synthesized and cloned into the Ubi‐MCS‐3FLAG‐CBh‐gcGFP‐IRES‐puromycin (GV492) backbone to generate expression vectors. For site‐directed mutagenesis, serine at position 211 in ABHD17C was replaced with alanine (S211A). In BCL6B, individual cysteine residues at positions 59, 177, 389, 417, and 442 were each substituted with serine (C59S, C177S, C389S, C417S, C442S), or all five cysteines to serines (5C‐5S). All mutants were constructed based on the overlap extension polymerase chain reaction (PCR) using the QuikChange Site‐directed Mutagenesis Kit (Stratagene) following the manufacturer's protocol. All constructs were verified by full‐length Sanger sequencing to confirm the intended mutations and exclude any unwanted alterations.

### 4.8 Western Blotting

Total proteins were extracted using a lysis buffer containing 2% SDS and supplemented with protease inhibitors. Protein concentrations were quantified, and the 30 µg of total protein per sample was separated on 8% or 12% SDS‐polyacrylamide gels. Following electrophoresis, the separated proteins were transferred onto nitrocellulose membranes (HATF00010, Millipore). The membranes were blocked with fat‐free milk for 1 h and then incubated with specific primary antibodies overnight at 4°C. After three washes with TBST, the membranes were incubated with a horseradish peroxidase (HRP)‐conjugated secondary antibody (1:5000, ZsBio) for 1 h at room temperature. After three additional TBST washes, protein bands were visualized using an enhanced chemiluminescence reagent kit (P1050, Applygen). The primary antibodies used are listed as follows: anti‐ABHD17C (1:1000, ab151040, Abcam), anti‐CD24 (1:1000, sc‐19585, Santa Cruz Biotechnology), anti‐BCL6B (1:1000, NBP1‐80434, Novus Biologicals), anti‐importin α (1:10 000, 10819‐1‐AP, Proteintech), anti‐importin β (1:10 000, 10077‐1‐AP, Proteintech), anti‐HA tag (1:2000, ab236632, Abcam), anti‐Flag tag (1:2000, 14793, Cell Signaling Technology), anti‐Ub tag (1:2000, 3936, Cell Signaling Technology), Anti‐Histone H3 (1:1000, 4620, Cell Signaling Technology), anti‐Streptavidin‐HRP (1:5000, SA00001‐0, Proteintech), and anti‐GAPDH (1:5000, 10494‐1‐AP, Proteintech).

### 4.9 Quantitative Real‐Time PCR (RT‐qPCR)

RT‐qPCR was performed as previously described [51, 55]. Briefly, total RNA was isolated from PC cells or tissues using TRIzol reagent (9108, Takara) according to the manufacturer's protocol. cDNA synthesis was performed using a reverse transcription kit (RR037A, Takara), followed by quantitative real‐time PCR with TB Green Premix Ex Taq (Tli RNaseH Plus, RR420A, Takara). GAPDH was used as the endogenous control, and fold‐changes were calculated by the 2^−ΔΔCT^ method. The sequences of the primers are listed as follows: ABHD17C: forward, 5’‐CTACTCGGGATACGGCGTCA‐3’; reverse, 5’‐AGAGGATAATGTTCTCGGGACTC‐3’. BCL6B: forward, 5’‐CTACGTCCGCGAGTTCACTC‐3’; reverse, 5’‐CCCGGAAAATTGAATAGAAG‐3’. CD24: forward, 5’‐AAGTAACTCCTCCCAGAGTACT‐3’; reverse, 5’‐GAGAGAGTGAGACCACGAAG‐3’. TNF‐α: forward, 5’‐GGCGTGTTCATCCGTTCTC‐3’; reverse, 5’‐CTTCAGCGTCTCGTGTGTTTCT‐3’. IFN‐γ: forward, 5’‐TCGGTAACTGACTTGAATGTCCA‐3’; reverse, 5’‐TCGCTTCCCTGTTTTAGCTGC‐3’. GAPDH: forward, 5’‐TGCCATCAATGACCCCTTC‐3’; reverse, 5’‐CATCGCCCCACTTGATTTTG‐3’.

### 4.10 Isolation of PBMCs and Generation of Macrophages

PBMCs were isolated from the whole blood by centrifugation at 400 × g for 20 min using human peripheral blood lymphocyte separation medium (7111011, Dakewe). The isolated PBMCs were then cultured for 7 days in the presence of 50 ng/mL GM‐CSF (RP00094, Abclonal) to generate macrophages.

### 4.11 Direct Co‐Culture Phagocytosis Assay

GFP^+^ PC cells were collected and labeled with pHrodo Red, SE (P36600, Thermo Fisher Scientific) at a dilution ratio of 1:30 000 in PBS for 1 h at 37°C according to the manufacturer's protocols. After labeling, the cells were washed three times with complete medium. Meanwhile, PBMC‐derived macrophages were harvested. Co‐culture suspensions containing 5 × 10^4^ macrophages and 1 × 10^6^ pHrodo Red‐labeled PC cells were prepared in serum‐free medium, supplemented with anti‐CD24 mAb (10 µg/mL; Clone SN3; NB100‐64861, Novus Biologicals), anti‐Siglec‐10 mAb (10 µg/mL; Clone 5G6; NBP3‐21494, Novus Biologicals), or an equivalent concentration of human IgG1 isotype control (Clone R1; A2051, Selleck). The plates were incubated for 6 h at 37°C and subsequently washed three times with PBS. Whole‐cell phagocytosis was assessed using an Olympus IX83 fluorescence microscope. Five random fields per well were imaged and the rate of internalized particles per cell was enumerated to quantify phagocytic activity.

### 4.12 Indirect Co‐Culture Induction Assay

To investigate PC cells on macrophage polarization, an indirect co‐culture assay was performed as previously described [56]. In brief, naïve macrophages (M0) differentiated from purified PBMCs were plated in the lower chamber of a Transwell insert (0.4 µm; 3460, Corning). PC cells were then seeded in the upper chamber, allowing for the exchange of soluble factors without direct cell contact. After 72 h of co‐culture, macrophages were harvested from the lower chamber and analyzed by FCM. CD163 (Clone RM3/1; 326506, BioLegend) was examined as a marker for M2‐like macrophages.

### 4.13 FCM Assay

Cells were harvested by standard enzymatic digestion, centrifuged, and resuspended in ice‐cold Cell Staining Buffer (420201, BioLegend). The cell density was adjusted to 5 × 10^5^ cells/mL, and 1 mL of cell suspension was aliquoted per sample. For human samples, Fc receptors were blocked with 5 µL Human TruStain Fc (422301, BioLegend) per 100 µL cell suspension for 10 min at room temperature. For mouse samples, Fc receptors were blocked with 0.25 µg TruStain Fc PLUS (anti‐mouse CD16/32; 156603, BioLegend) per 1 × 10^6^ cells in 100 µL for 10 min on ice. After centrifugation, cells were incubated with appropriately fluorochrome‐conjugated antibodies or IgG isotype controls for 30 min on ice in the dark. Following two washes with centrifugation, cells were resuspended in staining buffer and incubated on ice for 5 min before analysis. FCM was performed on an LSRFortessa analyzer (BD Biosciences), and the acquired data were analyzed using FlowJo software (V10). FCM antibodies and isotype controls are listed as follows: CD11b (Clone M1/70; 101230, BioLegend), CD45 (Clone 30‐F11; 103112, BioLegend), F4/80 (Clone W20065B; 111604, BioLegend), CD206 (Clone C068C2; 141717, BioLegend), CD24 (Clone ML5; 983602, BioLegend), mouse IgG1 isotype control (Clone MOPC‐21; BioLegend), and human IgG1 isotype control (Clone QA16A12; BioLegend).

### 4.14 FCM‐Based Phagocytosis Assay In Vitro

In vitro phagocytosis assays were conducted by co‐culturing PBMC‐derived macrophages and GFP^+^ PC cells at a ratio of 5 × 10^4^ macrophages to 1 × 10^5^ PC cells for 2 h at 37°C with 5% CO_2_. Cells were seeded in serum‐free medium into ultra‐low‐attachment 96‐well round‐bottom plates (7007, Corning). Anti‑CD24 mAb (Clone SN3; NB100‑64861, Novus Biologicals), anti‐Siglec‐10 mAb (Clone 5G6; NBP3‐21494, Novus Biologicals), or human IgG1 isotype control (Clone R1; A2051, Selleck) was added to the culture at a concentration of 10 µg/mL. ABD957 (HY‐142161, Med Chem Express) was added at 500 nm. After incubation, phagocytosis assays were terminated by transferring the plates to ice, followed by centrifugation at 400 × g for 5 min at 4°C. Samples were then incubated with fluorescence‐conjugated anti‐CD11b antibody (Clone M1/70; 101230, BioLegend) to identify macrophages by FCM analysis. Phagocytic activity was quantified as the percentage of CD11b^+^ GFP^+^ macrophages among total CD11b^+^ macrophages.

### 4.15 Cell Proliferation Assays

The proliferative capacity of PC cells in vitro was evaluated using the Cell Counting Kit‐8 (CCK‐8; CK04, Dojindo) assays as described previously [54, 57, 58]. Briefly, transfected cells were seeded into 96‐well plates and the absorbance was determined at 450 nm for 6, 24, 48, 72, and 96 h using a Spectra Max 190 microplate reader (Molecular Devices).

### 4.16 Mice

This study utilized 6‐ to 8‐week‐old female C57BL/6 mice (Beijing Vital River, Beijing, China) and NOD.Cg‐*Prkd*c^scid^ *Il2rg* ^em1Smoc^ (NSG) mice (The Jackson Laboratory, Bar Harbor, ME, USA). All animal experiments were conducted in accordance with protocols approved by the Animal Ethics Committee of the First Affiliated Hospital of University of Science and Technology of China [No.2024‐N(A)‐39]. The entire procedure was implemented in strict compliance with the specifications of the Regulations of the Administration of Laboratory Animals in China, with efforts made to minimize animal distress and pain, and to uphold the welfare of laboratory animals.

### 4.17 DMBA‐Induced Spontaneous Mouse Model of PC

A spontaneous PC model was induced with DMBA as previously described [51, 54]. Briefly, anesthetized C57BL/6 mice received a 1.5‐cm left‐flank incision to expose the spleen and distal pancreas. A 1‐cm‐diameter pocket was pre‐sutured at the pancreatic tail with CV‐7 sutures, and 1 mg of the potent carcinogen DMBA (D3254, Sigma–Aldrich) was deposited inside before the pouch was closed. The peritoneal cavity was inspected to ensure no leakage before wound closure. Two months after surgery, mice were euthanized under anesthesia and pancreatic lesions were evaluated.

### 4.18 Orthotopic PC‐CDX Model in NSG Mice

NSG mice were maintained under specific‐pathogen‐free conditions and acclimatized for 1 week after arrival to eliminate transport‐related stress. After a 6 h fast, mice were anesthetized, the left flank was depilated and disinfected, and a 1.5‐cm incision was made to expose the spleen and pancreatic tail. A suspension of 5 × 10^6^ transfected PC cells in 100 µL was slowly injected into the pancreatic tail. The injection site was gently pressed with a sterile cotton swab to prevent leakage. The pancreas and spleen were returned to the peritoneal cavity, and the incision was closed in layers with absorbable sutures. For antibody treatment, mice were administered intraperitoneal injections of either anti‐CD24 mAb (Clone SN3; NB100‐64861; Novus Biologicals) or mouse IgG1 isotype control (Clone MOPC‐21; A2106; Selleck) at a dose of 400 µg per mouse, twice weekly from day 7 to day 28 post‐engraftment. All mice were subjected to daily health monitoring. Following tumor engraftment, inspections were performed at least twice daily to ensure rapid identification and euthanasia when any humane endpoint was reached. Four weeks post‐engraftment, NSG mice were euthanized under anesthesia. Tumors were harvested, photographed, weighed, and measured for volume. Specimens were subsequently processed for FCM, qPCR, or paraffin embedding.

### 4.19 Macrophage Depletion Treatment In Vivo

Macrophage depletion in NSG mice was performed according to a previously published protocol [18]. Mice were administered 400 µg anti‐CSF1R antibody (A2159, Selleck) or PBS (vehicle) intraperitoneally every other day for 2 weeks before tumor engraftment and throughout the experimental period (Figure S7G). Successful depletion of tissue‐resident macrophages was confirmed by FCM‐based analysis of peritoneal lavage samples collected prior to tumor implantation (Figure S7H). Following confirmation of depletion, macrophage‐depleted and vehicle‐treated NSG mice were randomized and engrafted with either WT/ΔBCL6B cells with or with anti‐CD24 mAb as indicated (Figure S7G). Consistent macrophage depletion was monitored throughout the treatment period. (Figure S7H).

### 4.20 Survival Analysis of Orthotopic CDX Model

For survival analysis, the independent cohort was established under identical conditions to generate a survival dataset, including the same group assignments, mouse numbers, identically transfected PC cells, and equivalent antibody types/doses. Humane endpoints in this study were predefined as follows: (1) more than 20% loss of initial body weight; (2) evidence of metastasis to distant organs (e.g., lung); (3) persistent vocalization, trembling, or self‐mutilation; (4) inability to feed or drink independently, accompanied by signs of severe dehydration or malnutrition. Of note, postoperative day 60 was defined as the study endpoint. The survival curve for NSG mice was plotted based on the recorded survival status and time of death.

### 4.21 Single‐Cell Digestion of Tumor Tissues

Tumor tissues were processed as previously described [51]. Tumor masses were weighed and meticulously minced using surgical scissors. The minced tissue was then subjected to enzymatic digestion in a solution composed of RPMI‐1640 supplemented with 10% FBS, 0.5 mg/mL collagenase IV (C5138, Sigma–Aldrich), and 0.15 mg/mL DNase I (DN25, Sigma–Aldrich). The volume of digestion solution used was five times the tissue mass. Digestion was performed at 37°C in a water bath for 1 h, with gentle agitation at 10‐min intervals. After digestion, the mixture was diluted with a generous volume of complete culture medium and centrifuged. The pellet was resuspended and passed through a 40 µm cell strainer to remove undigested tissue fragments. Finally, the cell density was adjusted to 1 × 10^6^ cells/mL in ice‐cold Cell Staining Buffer (420201, BioLegend) for subsequent FCM analysis.

### 4.22 FCM‐Based Phagocytosis Assay In Vivo

Single‐cell suspensions from tumor tissue were prepared as described above. After Fc‐receptor blockade, cells were stained with fluorochrome‐conjugated antibodies or isotype controls for 30 min on ice, washed twice with FCM buffer, and resuspended in buffer containing 1 µg/mL 4’,6‐diamidino‐2‐phenylindole (DAPI; 422801, BioLegend) to exclude dead cells. Data were acquired on an LSRFortessa flow cytometer (BD Biosciences). Engulfing macrophages were defined as DAPI^−^ CD45^+^ F4/80^+^ CD11b^+^ GFP^+^; M2‐like macrophages as DAPI^−^ CD45^+^ F4/80^+^ CD11b^+^ CD206^+^.

### 4.23 IF Staining

Tissue sections from NSG mice or coverslips seeded with cells transfected with the indicated plasmids were fixed in 4% paraformaldehyde (G1110, Servicebio) for 15 min, permeabilized with 0.1% Triton X‐100 in PBS for 10 min, and blocked with 3% bovine serum albumin (BSA; ST025, Beyotime). Samples were incubated overnight at 4°C with primary antibodies against CD206 (1:200, 24595, Cell Signaling Technology), ABHD17C (1:500, ab151040, Abcam), and BCL6B (1:500, NBP1‐80434, Novus Biologicals), followed by 1 h with Alexa Fluor 488/594‐conjugated secondary antibodies (Solarbio, China). After washing, sections or coverslips were mounted in antifade medium containing DAPI (P0131, Beyotime). The fluorescence images were acquired using an inverted fluorescence microscope (Olympus IX83) or a confocal microscope (Olympus SpinSR10) and analyzed with Image J software (v1.48).

### 4.24 Co‐IP

Following transfection and treatment, PC cells were lysed on ice for 30 min using IP lysis buffer (P0013J, Beyotime) supplemented with protease inhibitors. The lysates were centrifuged at 12 000 × g for 20 min at 4°C. The supernatant was collected, and its protein concentration was determined using the BCA Protein Assay Kit (23225, Thermo Fisher Scientific) according to the manufacturer's instructions. An aliquot containing 150 µg of total protein was set aside as the “input” control. To the remaining supernatant, primary antibodies and protein A/G agarose beads (80106G, Thermo Fisher Scientific) were added, followed by overnight incubation at 4°C with gentle rotation. The beads were then washed three times with lysis buffer. Bound proteins were eluted with 1× SDS‐PAGE loading buffer and subsequently analyzed by immunoblotting.

### 4.25 Liquid Chromatography‐Tandem Mass Spectrometry (LC‐MS/MS)

Bead‐bound immunoprecipitates were sequentially washed, then resuspended in reaction buffer and incubated at 60°C for 1 h to accomplish protein denaturation, cysteine reduction, and alkylation. After dilution with an equal volume of H_2_O, the eluates were digested with 1 µg sequencing‐grade trypsin overnight at 37°C. The resultant peptides were desalted on in‐house‐packed SDB columns, vacuum‐dried, and stored at −20°C until analysis. Liquid chromatography combined with tandem mass spectrometry analysis was performed by Biogreen Biotechnology using a timsTOF Pro mass spectrometer (Bruker) coupled to a nanoElute system (Bruker Daltonics). Raw MS data from all samples were combined and processed with MaxQuant for protein identification and label‐free quantification. Subsequent bioinformatics analyses were conducted on the Lab‐4D platform, which integrates major public databases.

### 4.26 Molecular Docking

The amino‐acid sequences of human ABHD17C (UniProt ID: Q6PCB6) and BCL6B (UniProt ID: Q8N143) were retrieved from the UniProt database. Rigid‐body protein‐protein docking was performed with GRAMM using global scanning and default parameters. Among the generated poses, the lowest‐energy complex was selected and visualized with PyMOL.

### 4.27 PLA

PLA was employed to detect the intracellular interaction between ABHD17C and BCL6B. Mia paca‐2 or Panc‐1 cells were co‐transfected with ABHD17C and BCL6B expression constructs. Cells cultured on 12‐mm coverslips were fixed, permeabilized, blocked, and incubated overnight at 4°C with primary antibodies under the same conditions used for the above‐mentioned IF staining. After three washes with PBS, Duolink PLA probes were incubated for 1 h at 37°C. Hybridization, ligation, and rolling‐circle amplification were performed according to the manufacturer's instructions using the Duolink in Situ Detection Kit (DUO92014, Sigma–Aldrich). Nuclei were counterstained with DAPI. Amplified products were visualized as discrete green fluorescent puncta with a confocal microscope (Olympus SpinSR10). Negative controls lacked either one or both primary antibodies.

### 4.28 ABE Assays

BCL6B palmitoylation was assessed using the IP‐ABE Palmitoylation Kit for WB (AM10313, AIMSMASS) according to the manufacturer's protocols. The assay procedure consisted of blocking, reduction, labeling, elution, and detection steps. Briefly, PC cells were harvested and lysed, followed by overnight immunoprecipitation at 4°C using antibody‐coupled beads against BCL6B or Flag tag. Unmodified cysteine residues were blocked by treatment with N‐ethylmaleimide (NEM; 23030, Thermo Fisher Scientific) for 30 min. Beads were then washed and divided into two groups: one treated with 1 M HAM (26103, Thermo Fisher Scientific) for 1 h at room temperature to cleave thioester bonds (+HAM), and the other incubated in parallel without HAM (−HAM) as a negative control. After washing, both groups were labeled with thiol‐reactive biotin for 1 h at room temperature. Beads were then resuspended in SDS‐PAGE loading buffer and boiled for 10 min. Palmitoylation levels were detected by immunoblotting with streptavidin‐HRP.

### 4.29 Click‐iT Identification of BCL6B Palmitoylation

After plasmid transfection, PC cells were incubated with 100 mm of Click‐iT palmitic acidazide (C10265, Thermo Fisher Scientific) at 37°C for 6 h. The cells were then washed with PBS and lysed on ice for 20 min. Following sonication and clarification (12 000 × g, 4°C, 10 min), protein concentration was determined by BCA assay. The click reaction was performed using the Click‐iT Protein Reaction Buffer Kit (C10276, Thermo Fisher Scientific) to conjugate biotin alkyne to the labeled palmitoylated proteins. Biotin alkyne‐azide‐palmitic‐protein complex was captured with streptavidin and subsequently analyzed by immunoblotting for BCL6B.

### 4.30 Ubiquitination Assays

PC cells grown to approximately 80% confluence were transfected with the indicated plasmids. After 48 h, the cells were treated with 10 µm MG132 (M8699, Sigma–Aldrich) for 6 h. Cells were then lysed in RIPA buffer supplemented with protease inhibitors. Following centrifugation at 12 000 × g for 20 min at 4°C, the supernatant was collected, and protein concentration was determined using the BCA Protein Assay Kit (23225, Thermo Fisher Scientific) according to the manufacturer's protocols. A total of 150 µg protein from each sample was retained as the “input” control. Equal amounts of protein lysate were incubated with protein A/G magnetic beads (80106G, Thermo Fisher Scientific) that had been pre‐coupled with the target antibody overnight. The immunoprecipitated proteins were eluted in 1× SDS‐PAGE loading buffer and subjected to immunoblotting analysis.

### 4.31 Cytosolic/Nuclear Fractionation

Nuclear and cytoplasmic fractions were prepared with the PARIS kit (AM1921, Thermo Fisher Scientific) according to the manufacturer's protocols. After a 6‐h treatment with 10 µm MG132 (M8699, Sigma–Aldrich), cells from a 10‐cm dish were harvested, resuspended in pre‐chilled fractionation buffer, and then centrifuged at 500 × g for 5 min at 4°C. The supernatant was collected as the cytoplasmic fraction; the pellet was lysed in cell‐disruption buffer to yield the nuclear extract. Nuclear and cytoplasmic lysates were processed for protein extraction.

### 4.32 Dual Luciferase Reporter Assay

Cells were seeded in 24‐well plates and co‐transfected with either WT or mutant CD24 (CD24‐mut) constructs along with the Renilla luciferase plasmid pRL‐TK for 24 h. Following transfection, cells were lysed according to the manufacturer's instructions using the lysis buffer provided in the Dual‐Luciferase Reporter Assay Kit (E1910, Promega). Luminescence was measured on an Infinite M200 PRO luminometer (Tecan). Firefly luciferase activity was normalized to Renilla values.

### 4.33 ChIP Analysis

ChIP‐qPCR was performed in PC cells using the Pierce Agarose ChIP Kit (26156, Thermo Fisher Scientific) according to the manufacturer's protocol. Cells were cross‐linked with 1% formaldehyde for 10 min at room temperature, quenched with 125 mm glycine, and DNA was sheared into fragments with micrococcal nuclease at 37°C for 20 min. Cross‐linked protein‐DNA complexes were immunoprecipitated overnight at 4°C with rabbit anti‐BCL6B (NBP1‐80434, Novus Biologicals) or rabbit IgG (A7016, Beyotime). After sequential washes, de‐cross‐linking, and proteinase K digestion, DNA was purified with spin columns and quantified by qPCR using gene‐specific primers.

### 4.34 Statistical Analysis

All statistical analyses were conducted using SPSS Statistics 22.0 (IBM, USA), and graphs were generated with GraphPad Prism 7.0 (Dotmatics, La Jolla, USA). Each experiment was performed at least in triplicate, with data presented as mean ± standard deviation (SD). Statistical methods were selected based on data distribution characteristics. The Shapiro‐Wilk test and Q‐Q plot were used to assess the normality of the data. Levene's test was employed to test for homogeneity of variances. For two‐group analyses, parametric data were assessed using Student's *t*‐test, while non‐parametric data were analyzed with the Mann‐Whitney *U* test. For comparisons among multiple groups, one‑ or two‑way ANOVA was applied, followed by Dunnett's, Tukey's, or Bonferroni's post‑hoc tests. Linear correlations between variables were evaluated using Spearman's correlation. Associations between categorical variables were assessed using Pearson's χ^2^ test. Survival curves were generated using the Kaplan–Meier method with Log‐rank (Mantel‐Cox) tests for comparison. Multivariate survival analysis was conducted using the Cox proportional hazards model. A two‐tailed *p*‐value < 0.05 was considered statistically significant.

## Author Contributions

Y.Z., D.C., and F.M. contributed equally to this work. Y.Z. designed the study, performed most experiments, analyzed data, and drafted the manuscript; D.C. and F.M. performed the animal experiments, data analysis, and interpretation; X.Z., L.Z., L.W., and H.H. conducted a part of the molecular biology experiments, clinical data, and bioinformatics analysis, and edited the manuscript; L.L., H.Y., and J.W. contributed to the design, coordination, and supervised progress. All authors have read and approved the final version of the manuscript.

## Conflicts of Interest

The authors declare no conflicts of interest.

## Supporting information




**Supporting File**: advs75757‐sup‐0001‐SuppMat.docx.

## Data Availability

The data that support the findings of this study are available from the corresponding author upon reasonable request.
