# Supplementary material for: ABHD17C‐Mediated S‐Depalmitoylation of BCL6B Enhances CD24 Transcription to Resist Macrophage Phagocytosis in Pancreatic Cancer
Source: Adv Sci (Weinh). 2026 May 19;13(45):e75757. doi: 10.1002/advs.75757 (PMC13336133; doi:10.1002/advs.75757)
Supplement: Supplementary file 1 [file ADVS-13-e75757-s001.docx]

**Supporting Information**

**ABHD17C-mediated S-depalmitoylation of BCL6B enhances CD24 transcription to resist macrophage phagocytosis in pancreatic cancer**

*Yalu Zhang^#^, Di Cui^#^, Fanzheng Meng^#^, Xu Zhu, Liang Zhu, Liang Wu, Haifeng Hu, Jizhou Wang*, Hanhui Yao*, Lianxin Liu**

1. Supplementary Figures and Figure Legends………….…………………Page 2-13

2. Supplementary Tables………………….……………….……………Page 14-16

**1. Supplementary Figures and Figure Legends**

**Figure S1.** Aberrant ABHD17C upregulation predicts unfavorable prognosis in PC. (A) The Human Protein Atlas was used to evaluate ABHD17C expression between pancreatic cancer (PC) and normal pancreatic tissues. Scale bar, 200μm. (B) Analysis of single-cell RNA sequencing data (CRA001160) from PC tissue was performed to investigate ABHD17C expression across distinct cellular subpopulations within the tumor microenvironment, including malignant PC cells (red circle), normal ductal cells (green circle), acinar cells, endocrine cells, fibroblasts, immune cells, and other cell types. (C-E) TIMER, LinkedOmics, and TISIDB databases were used to analyze the impact of ABHD17C expression on OS in patients with PC. (F) The expression level of ABHD17C was analyzed in PC tissues across different pathological stages using the GEPIA web tool. (G,H) The differential expression of ABHD17C was investigated across different tumor sizes and clinical stages in PC using the LinkedOmics database. (I) ABHD17C expression differences were compared among normal pancreatic tissue, primary PC tumors, liver metastases, and lung metastases using the GSE71729 dataset. Abbreviation: PC, pancreatic cancer; TIMER, Tumor Immune Estimation Resource; TISIDB, Tumor-Immune System Interaction Database; OS, overall survival; GEPIA, Gene Expression Profiling Interactive Analysis; Met, metastatic. Each error bar in F-I represents the mean ± SD; **p* < 0.05, ***p* < 0.01, ****p* < 0.001, *****p* < 0.0001. Statistical analysis was performed using the log-rank test (C-E), a one-way ANOVA with Tukey’s post-hoc test (F,I), or a one-tail Mann-Whitney *U* test (G,H).

**Figure S2.** Subgroup survival analyses of ABHD17C in PC patients stratified by gender, age, histological grade, tumor size, perineural invasion, microvascular invasion, and serum CA199 level. The sample sizes for each group and the *p*-values as indicated. Survival curves were compared using the Log-rank test.

**Figure S3.** ABHD17C confers macrophage phagocytic resistance via potentiation of CD24 signaling. (A) Left panel: representative immunohistochemistry (IHC) images depicting tumor-associated macrophage (TAM) infiltration (F4/80) in the primary pancreatic cancer (PC) and lung metastases of a DMBA-induced spontaneous PC model. Right panel: representative IHC images of TAM infiltration (CD68) in the primary PC lesions from patients. Red bar: 100μm; black bar: 50μm. (B) TIMER-database analysis of the correlation between ABHD17C expression and TAM infiltration in PC. (C-E) The correlation between ABHD17C and CD24 expression was analyzed using LinkOmics, GEPIA, and TIMER web tools. (F) Quantification of ABHD17C protein expression by Western blot in the HPNE cell line and seven PC cell lines. (G-I) The mRNA, protein, and cell surface levels of CD24 following ABHD17C knockout were detected by qPCR, Western blot, and flow cytometry, respectively. (J) Representative phagocytosis images of peripheral blood mononuclear cell (PBMC)-derived macrophages co-cultured with pHrodo-red⁺ GFP⁺ Mia paca-2 cells, with or without ABHD17C-KO. Red puncta inside macrophages indicate engulfed tumor cells. Scale bar, 100μm. (K) Representative flow cytometry (FCM) plots showing macrophage phagocytosis of Mia paca-2 cells, with or without ABHD17C-KO. CD11b^+^ GFP^+^ cells were defined as engulfed cells. (L) Representative images of phagocytosis assays. PBMC-derived macrophages were co-cultured with pHrodo-red⁺ GFP⁺ SW1990 or Panc-1 cells. Tumor cells had been transfected with ABHD17C-plasmid or empty vector and pre-incubated with IgG control or anti-Siglec-10 mAb. Red puncta inside macrophages indicate engulfed tumor cells. Scale bar, 100μm. (M) FCM-based measurement showing phagocytosis of SW1990 or Panc-1 cells, transfected with ABHD17C-plasmid or empty vector, and pre-treated with IgG control or anti-Siglec-10 mAb. Cells double-positive for CD11b and GFP were defined as engulfed cells. Abbreviation: *Prim.*, primary; *Met.*, metastatic; PC, pancreatic cancer; TPM, transcripts per million; *Rel.*, relative; Ctr, control; GEPIA, Gene Expression Profiling Interactive Analysis; TIMER, Tumor Immune Estimation Resource. Data are the mean ± SD of n = 3 (F,G,J-M) independent biological replicates; **p* < 0.05, ***p* < 0.01, ****p* < 0.001, *****p* < 0.0001; *NS*, not significant. Statistical analysis was performed using two-tailed Spearman’s test (B-E), or a one-way ANOVA with Dunnett’s post-hoc test (F,G,J,K) or with Tukey’s post-hoc test (L,M).

**Figure S4.** Gating strategy, cell proliferation assays and phagocytosis assays. (A) Gating strategy for *in vitro* phagocytosis assay. Following debris and doublet removal, phagocytosis was defined as the percentage of DAPI^−^ CD11b^+^ FITC^+^ events out of all DAPI^−^CD11b^+^ events. Numbers indicate frequency of events out of previous gate. (B) Gating strategy for *in vitro* M2-like polarization. Following debris and doublet removal, M2-like polarization was defined as the percentage of DAPI^−^ CD163^+^ events out of all DAPI^−^ events. Numbers indicate frequency of events out of previous gate. (C) The CCK-8 proliferation assay was employed to evaluate the proliferative capacity of Mia PaCa-2 cells following ABHD17C knockout and of SW1990 cells following ABHD17C overexpression. Absorbance was measured at 450 nm after 6, 24, 48, 72 and 96h. (D) *In vivo* gating strategy for tumor-associated macrophage (TAM) phagocytosis of pancreatic cancer (PC) cells. After exclusion of debris and doublets, phagocytic TAMs were quantified as the percentage of DAPI⁻ CD11b⁺ F4/80⁺ GFP⁺ events within the total DAPI⁻ CD11b⁺ F4/80⁺ population. M2-like TAMs were identified as DAPI⁻ CD11b⁺ F4/80⁺ CD206⁺ cells. All numeric values represent the frequency of events relative to the preceding gate. (E) The protein levels of ABHD17C and BCL6B were detected in ABHD17C-knockout Mia paca-2 cells, as well as in ABHD17C-overexpressing SW1990 and Panc-1 cells. (F) Phagocytosis assays were performed by co-culturing PBMC-derived macrophages with pHrodo-red⁺ SW1990 or Panc-1 cells transfected with ABHD17C, BCL6B, or both. Red puncta inside macrophages indicate engulfed SW1990 or Panc-1 cells. Scale bar, 100μm. Abbreviation: DAPI, 4’,6-diamidino-2-phenylindole; PBMCs, peripheral blood mononuclear cells; PC, pancreatic cancer; OD, optical density; Ctr, control. Data are the mean ± SD of n = 3 (C,F) independent biological replicates; **p* < 0.05, ***p* < 0.01, ****p* < 0.001; *NS*, not significant. Statistical analysis was performed using a two-way repeated measures ANOVA with Dunnett’s post-hoc test (C) or a one-way ANOVA with Tukey’s post-hoc test (F).

**Figure S5.** ABHD17C-mediated depalmitoylation of BCL6B promotes its degradation in a ubiquitination manner. (A) The predicted palmitoylation sites of BCL6B from CSS-Palm 4.0 software. (B) BCL6B palmitoylation at Cys442 was predicted using CSS-Palm 4.0 and the resulting BCL6B sequences were aligned to assess conservation across species. (C,D) The degradation of BCL6B in Mia paca-2 cells treated with or without 2-BP was detected by cycloheximide (CHX)-chase assay in the presence of proteasomal inhibitors (MG132 and Carfilzomib) and lysosomal inhibitors (NH_4_Cl and Chloroquine). Quantification of the intensity assessed by the relative level of BCL6B remaining. (E,F) The degradation of BCL6B in Panc-1 cells with or without ABHD17C overexpression was assessed by CHX-chase assay in the presence of proteasomal inhibitors (MG132 and Carfilzomib) and lysosomal inhibitors (NH_4_Cl and Chloroquine). Quantification of the intensity evaluated by the relative level of BCL6B remaining. (G) The ubiquitination levels of BCL6B were detected in Mia paca-2 cells transfected with Flag-BCL6B, with or without ABHD17C knockout, followed by MG132 treatment. (H) The ubiquitination levels of BCL6B were assessed in Panc-1 cells transfected with Flag-BCL6B WT/C442S plasmids followed by MG132 treatment. Abbreviation: CSS, clustering and scoring strategy; CHX, cycloheximide; DMSO, dimethyl sulfoxide; Ctr, control; Ub, ubiquitin; IP, immunoprecipitation; WT, wild-type. Data are the mean ± SD of n = 3 (D,F) independent biological replicates. Statistical analysis was performed using a two-way repeated measures ANOVA with Bonferroni’s post-hoc test (D,F).

**Figure S6.** ABHD7C-mediated BCL6B depalmitoylation inhibits its nuclear translocation by disrupting the interaction with importin-α/β. (A) Representative immunofluorescence (IF) images of Panc-1 cells infected with empty vector or ABHD17C plasmids followed by MG132 treatment. Scale bar, 10 μm**.** (B) Statistical analysis of BCL6B fluorescence intensity in the nuclear and cytoplasmic compartments of the indicated groups. (C) Western blot analysis for cytosolic and nuclear fractions from Panc-1 cells transfected with empty vector or ABHD17C plasmids followed by MG132 treatment. (D) Under MG132 treatment, representative IF images of Panc-1 cells transfected with ABHD17C WT or S211A mutation, or with ABHD17C WT combined with ABD957 treatment. Scale bar, 10 μm**.** (E) Statistical analysis of BCL6B fluorescence intensity in the nuclear and cytoplasmic compartments of the indicated groups. (F) Under MG132 treatment, fractionation analysis of BCL6B in Panc-1 cells transfected with ABHD17C WT or S211A mutation, or with ABHD17C WT + ABD957 treatment. (G) Representative IF images of Panc-1 cells transfected with BCL6B WT/C442S followed by MG132 treatment. Scale bar, 10 μm**.** (H) Statistical analysis of BCL6B fluorescence intensity in the nuclear and cytoplasmic compartments of the indicated groups. (I) Fractionation analysis of BCL6B in Panc-1 cells transfected BCL6B WT/C442S followed by MG132 treatment. (J,K) Importin α/β and Flag were analyzed by Western blot after immunoprecipitation with anti-Flag antibody from Mia paca-2 and SW1990 cells transfected with Flag-WT or Flag-C442S. Abbreviation: DAPI, 4’,6-diamidino-2-phenylindole; WCL, whole cell lysate; WT, wild-type; IP, immunoprecipitation. Data are the mean ± SD of n = 3 (B,E,H) independent biological replicates; ***p* < 0.01. Statistical analysis was performed using an unpaired two-tailed Student’s *t* test (B,H) or a one-way ANOVA with Dunnett’s post-hoc test (E).

**Figure S7.** BCL6B directly binds to the CD24 promoter and negatively regulates its expression. (A,B) Flow cytometry (FCM) for detecting the expression of CD24 on the cell membrane in BCL6B-overexpressing Mia paca-2 and Panc-1 cells, as well as in BCL6B-KO SW1990 cells. (C) Locations of ChIP-PCR primers [Site 1 (-883 ~ -867), 2 (-741 ~ -725), 3 (-238 ~ -222)] at the CD24 promoter. Transcription start site (TSS) was designated as nucleotide + 1. (D) JASPAR was used to predict the binding sites between BCL6B and the promoter of CD24. The mutated sequences of MUT1, MUT2, and MUT3 are presented. (E) Representative images of phagocytosis assays. Peripheral blood mononuclear cell (PBMC)-derived macrophages were co-cultured with pHrodo-red⁺ SW1990 cells with or with BCL6B-KO, and pre-incubated with IgG control or anti-Siglec-10 mAb. Red puncta inside macrophages indicate engulfed SW1990 cells. Scale bar, 100 μm. (F) Representative FCM plots showing phagocytosis of GFP^+^ SW1990 cells with or with BCL6B-KO, and pre-treated with IgG control or anti-Siglec-10 mAb. CD11b^+^ GFP^+^ were defined as engulfed cells. (G) Schematic illustration of macrophage-depleted (anti-CSF1R mAb) and vehicle-treated (PBS) orthotopic pancreatic CDX modeling using BCL6B-WT/KO cells, followed by treatment with anti-CD24 mAb or IgG1 control in NSG mice. (H) Left panel: representative flow cytometry plots showing tissue-resident macrophages out of total live cells in vehicle- vs. anti-CSF1R-treated mice; numbers indicate frequency of CD11b^+^ F4/80^+^ macrophage (Tumor-associated macrophage, TAM) events out of total live events. Right panel: the percentage of TAMs out of total live cells in vehicle- (n = 4) vs. anti-CSF1R-treated mice (n = 4) as measured by flow cytometry at 3 days before tumor engraftment and throughout the experimental period. ICGC, International Cancer Genome Consortium; TSS, transcription start site; WT, wild-type; MUT, MUT, mutation site; i.p., intraperitoneal; QOD, every other day; PC, pancreatic cancer; CDX, cell derived xenograft; BIW, twice a week; PBS, phosphate-buffered saline; mAb, monoclonal antibody. Data are the mean ± SD of n = 3 (A,B,E,F) and n = 4 (H) independent biological replicates; **p* < 0.05, ***p* < 0.01, ****p* < 0.001; *NS*, not significant. Statistical analysis was performed using an unpaired two-tailed Student’s *t* test (A,B), a one-way ANOVA with Tukey’s post-hoc test (E,F), or two-way repeated measures ANOVA with Bonferroni’s post hoc test (H).

**Figure S8.** Catalytically dead S211A mutant fails to rescue the ABHD17C-KO or BCL6B-OE phenotype. (A) Representative images of multiplex immunofluorescence staining in PC tissues with DAPI, ABHD17C, BCL6B and CD24. Scale bar, 20 μm. (B) Western blot analysis for the expression of CD24, BCL6B, and Flag in ABHD17C-konockout Mia paca-2 cells infected with empty vector, Flag-ABHD17C WT/S211A plasmids. (C,D) The degradation of BCL6B and CD24 was analyzed by CHX-chase assay in ABHD17C-KO Mia paca-2 cells or BCL6B-OE Panc-1 cells that were transfected with empty vector control, ABHD17C-WT, or ABHD17C-S211A. (E,G) Representative images of phagocytosis by macrophages co-cultured with pHrodo Red-labeled ABHD17C-KO Mia paca-2 cells or BCL6B-OE Panc-1 cells that were transfected with empty vector control, ABHD17C-WT, or ABHD17C-S211A. Red puncta inside macrophages indicate engulfed tumor cells. Scale bar, 100μm. (F,H) Flow cytometric analysis of phagocytosis in ABHD17C-KO Mia paca-2 cells or BCL6B-OE Panc-1 cells after transfection with empty vector, ABHD17C-WT, or the catalytically inactive ABHD17C-S211A. The frequency of phagocytosis events out of all macrophages among different groups. CD11b^+^ GFP^+^ cells were defined as engulfed cells. Data are the mean ± SD of n = 3 (E-H) independent biological replicates; ***p* < 0.01; *NS*, not significant. Statistical analysis was performed using a one-way ANOVA with Tukey’s post-hoc test (E-H).

**2. Supplementary Tables**

| **Table S1. Relationship between ABHD17C expression and clinicopathological characteristics of patients with pancreatic cancer (N = 97)** | | | | |
| --- | --- | --- | --- | --- |
| **Parameters** | **Total** | **ABHD17C expression** | | ***p*-value** |
|  |  | **Low (n=47)** | **High (n=50)** |  |
| **Age (years)** |  |  |  | 0.763 |
| <60 | 49 | 23 | 26 |  |
| ≥60 | 48 | 24 | 24 |  |
| **Gender** |  |  |  | 0.544 |
| Female | 61 | 31 | 30 |  |
| Male | 36 | 16 | 20 |  |
| **Histological grade** |  |  |  | 0.784 |
| G1 | 26 | 12 | 14 |  |
| G2-G3 | 71 | 35 | 36 |  |
| **Tumor size (cm)** |  |  |  | **0.014*** |
| <4 | 79 | 43 | 36 |  |
| ≥4 | 18 | 4 | 14 |  |
| **Lymph node metastasis** |  |  |  | **0.011*** |
| Negative | 37 | 24 | 13 |  |
| Positive | 60 | 23 | 37 |  |
| **TNM stage** |  |  |  | **0.010*** |
| Ⅰ-ⅡA | 33 | 22 | 11 |  |
| ⅡB-Ⅳ | 64 | 25 | 39 |  |
| **CA199** |  |  |  | 0.076 |
| Normal | 27 | 17 | 10 |  |
| Elevated | 70 | 30 | 40 |  |
| **Perineural invasion** |  |  |  | 0.464 |
| Negative | 85 | 40 | 45 |  |
| Positive | 12 | 7 | 5 |  |
| **Macrovascular invasion** |  |  |  | 0.064 |
| Negative | 77 | 41 | 36 |  |
| Positive | 20 | 6 | 14 |  |
| **p* < 0.05 | | | | |

| **Table S2. Univariate and multivariate analyzes for prognostic factors of PC** | | | | | |
| --- | --- | --- | --- | --- | --- |
| **Parameters** | **Univariate analysis** | |  | **Multivariate analysis** | |
|  | **HR (95% CI)** | ***p*-value** |  | **HR (95% CI)** | ***p*-value** |
| Age (years): ≥60 vs <60 | 0.658 (0.410-1.054) | 0.082 |  | — | — |
| Gender:  Male vs female | 1.037 (0.638-1.685) | 0.884 |  | — | — |
| Histological grade: G1 vs G2-3 | 0.454 (0.275-0.751) | **0.002*** |  | 0.447 (0.267-0.746) | **0.002*** |
| Tumor size (cm): ≥4 vs < 4 | 2.226 (1.275-3.885) | **0.005*** |  | 2.203 (1.195-4.063) | **0.011*** |
| Lymph node metastasis: Positive vs negative | 3.073 (1.813-5.210) | **<0.001*** |  | 2.425 (0.577-10.185) | 0.226 |
| TNM stage: ⅡB-Ⅳ vs Ⅰ-ⅡA | 2.927 (1.700-5.041) | **<0.001*** |  | 1.108 (0.253-4.861) | 0.829 |
| CA199: Elevated vs normal | 3.797 (1.930-7.471) | **<0.001*** |  | 3.071 (1.506-6.260) | **0.002*** |
| Perineural invasion: Positive vs negative | 1.416 (0.808-2.481) | 0.224 |  | — | — |
| Macrovascular invasion: Positive vs negative | 1.006 (0.481-2.103) | 0.988 |  | — | — |
| ABHD17C expression: High vs low | 2.078 (1.280-3.373) | **0.003*** |  | 1.396 (1.069-2.075) | **0.018*** |
| Abbreviations: PC, pancreatic cancer; HR, hazard ratio; vs, versus; CI, confidence interval; **p* < 0.05. | | | | | |

| **Table S3. The prognostic relevance of ABHD17C expression in PC subgroups in which the poorer OS of patients is significantly associated with high ABHD17C expression** | | | |  |
| --- | --- | --- | --- | --- |
| **Subgroups** | **HR** | **95% CI** | ***p*-value** |  |
|  |  |  |  |  |
| Age ≥ 60 years | 2.998 | 1.439-6.246 | 0.0034 |  |
| Male | 2.354 | 1.265-4.380 | 0.0069 |  |
| Histological grade, G2-G3 | 2.366 | 1.310-4.271 | 0.0043 |  |
| Tumor size, ＜4cm | 2.121 | 1.205-3.735 | 0.0092 |  |
| Perineural invasion, negative | 1.859 | 1.067-3.239 | 0.0286 |  |
| Macrovascular invasion, negative | 2.123 | 1.272-3.543 | 0.0040 |  |
| CA199, normal | 4.381 | 1.119-17.16 | 0.0339 |  |

Abbreviations: PC, pancreatic cancer; OS, overall survival; HR, hazard ratio; vs, versus; CI, confidence interval.
